# Mitochondrion-targeted carboxymethyl chitosan hybrid nanoparticles loaded with Coenzyme Q10 protect cardiac grafts against cold ischaemia‒reperfusion injury in heart transplantation

**DOI:** 10.1186/s12967-023-04763-7

**Published:** 2023-12-20

**Authors:** Shun Yuan, Yanjia Che, Zhiwei Wang, Kai Xing, Xiaoping Xie, Yuanyang Chen

**Affiliations:** 1 Department of Cardiovascular Surgery, Renmin Hospital of Wuhan University, 238# Jiefang Road, Wuhan, 430000 Hubei People’s Republic of China; 2 Central Laboratory, Renmin Hospital of Wuhan University, Wuhan, China; 3 Key Laboratory of Biomedical Polymers of Ministry of Education, Department of Chemistry, Wuhan University, Wuhan, 430072 Hubei People’s Republic of China

**Keywords:** Heart transplantation, Cold I/R injury, Mitochondrion-targeted, Carboxymethylchitosan hybrid nanoparticles, CoQ10, Oxidative damage

## Abstract

**Background:**

Heart transplantation (HT) has been approved as an optimal therapeutic regimen for patients with terminal-stage cardiac failure. However, cold ischaemia‒reperfusion (I/R) injury remains an unavoidable and outstanding challenge, which is a major factor in early graft dysfunction and an obstacle to long-term survival in HT. Cold I/R injury induces cardiac graft injury by promoting mitochondrial dysfunction and augmenting free radical production and inflammatory responses. We therefore designed a mitochondrion-targeted nanocarrier loaded with Coenzyme Q10 (CoQ10) (CoQ10@TNPs) for treatment of cold I/R injury after cardiac graft in a murine heterotopic cardiac transplantation model.

**Methods:**

Hybrid nanoparticles composed of CaCO_3_/CaP/biotinylated-carboxymethylchitosan (CaCO_3_/CaP/BCMC) were synthesized using the coprecipitation method, and the mitochondria-targeting tetrapeptide SS31 was incorporated onto the surface of the hybrid nanoparticles through biotin-avidin interactions. Transmission electron microscopy (TEM) and dynamic light scattering (DLS) analysis were used for characterisation. In vitro, the hypoxia-reoxygenation model of H9c2 cells was employed to replicate in vivo cold I/R injury and treated with CoQ10@TNPs. The impact of CoQ10@TNPs on H9c2 cell injury was assessed by analysis of oxidative damage and apoptosis. In vivo, donor hearts (DHs) were perfused with preservation solution containing CoQ10@TNPs and stored in vitro at 4 °C for 12 h. The DHs were heterotopically transplanted and analysed for graft function, oxidative damage, apoptosis, and inflammatory markers 1 day post-transplantation.

**Results:**

CoQ10@TNPs were successfully synthesized and delivered CoQ10 to the mitochondria of the cold ischaemic myocardium. In vitro experiments demonstrated that CoQ10@TNPs was taken up by H9c2 cells at 4 °C and localized within the mitochondria, thus ameliorating oxidative stress damage and mitochondrial injury in cold I/R injury. In vivo experiments showed that CoQ10@TNPs accumulated in DH tissue at 4 °C, localized within the mitochondria during cold storage and improved cardiac graft function by attenuating mitochondrial oxidative injury and inflammation.

**Conclusions:**

CoQ10@TNPs can precisely deliver CoQ10 to the mitochondria of cold I/R-injured cardiomyocytes to effectively eliminate mitochondrial reactive oxygen species (mtROS), thus reducing oxidative injury and inflammatory reactions in cold I/R-injured graft tissues and finally improving heart graft function. Thus, CoQ10@TNPs offer an effective approach for safeguarding cardiac grafts against extended periods of cold ischaemia, emphasizing the therapeutic potential in mitigating cold I/R injury during HT. These findings present an opportunity to enhance existing results following HT and broaden the range of viable grafts for transplantation.

**Supplementary Information:**

The online version contains supplementary material available at 10.1186/s12967-023-04763-7.

## Introduction

Heart transplantation (HT) is the most widely accepted treatment for individuals suffering from terminal-stage cardiac failure [1]. According to the latest statistics, despite the presence of 7386 individuals on the waiting list for HT and the unfortunate fact that more than 500 patients passed away while awaiting a transplant, only approximately 3715 heart transplants were performed in the United States in 2020 [2]. This imbalance is due to the scarcity of available donor hearts (DHs) that are predicted to function effectively after procurement, preservation, and engraftment at transplantation. In an era of DH shortage, alleviating DH damage to improve the condition of DHs is an indispensable tool for physicians specializing in organ transplantation to increase organ utilization and address this dilemma.

Cold ischaemia‒reperfusion (I/R) injury, a multifactorial inflammatory event during DH procurement, preservation, and engraftment, is an inevitable problem in HT. Cold I/R injury is closely associated with early graft failure and enhances allograft immunogenicity, triggering and promoting acute and chronic rejection [3]. Furthermore, cold I/R injury exacerbates allograft damage, which imposes restrictions on the utilization of marginal DHs, ultimately exacerbating the imbalance between the number of patients waiting for transplant and available donors. Currently, the typical duration of cold ischaemia time (CIT) in DH ranges from 4 to 6 h, and a CIT equal to or exceeding 4 h is significantly linked to reduced survival rates compared to a CIT less than 4 h [4]. Therefore, developing new technical preservation protocols to mitigate cold I/R injury to DH could improve transplantation outcomes and attenuate immunological rejection. Furthermore, these methods could facilitate the safe expansion of presently acceptable CITs to address the scarcity of available DHs.

Although the exact mechanism of cold I/R injury requires further elucidation, previous studies have verified that cold I/R injury is closely associated with mitochondrial oxidative stress caused by reactive oxygen species (ROS) in reperfusion and simultaneous reoxygenation [5]. Abnormal generation of ROS and the buildup of calcium in mitochondria during ischaemia activate the mitochondrial permeability transition pore (MPTP), leading to mitochondrial structure destruction and dysfunction [6]. This injury appears during ischaemia and continues into reperfusion, thus seriously decreasing cardiac graft function and survival. Moreover, mitochondrial ROS (mtROS) and injured mitochondrial elements such as oxidized mitochondrial DNA (mtDNA) can activate inflammasomes, thus triggering an innate immune response and accelerating rejection [7].

Traditional antioxidants have not been effective in preventing mitochondrial oxidative damage in clinical cardiac cold I/R injury, mainly because of their limited ability to penetrate mitochondria [3]. In addition, conventional antioxidants are not released slowly and continuously in the transplanted heart in 24 h. In contrast to conventional medicine formulas, nanocarrier drug delivery systems offer improved therapeutic efficacy and the ability to achieve targeted delivery. By incorporating targeting ligands, drug delivery systems can selectively transport the loaded medication to desired locations, maximizing therapeutic efficacy while minimizing potential side effects [8]. To make use of these advantages, we designed a mitochondrion-targeted nanocarrier loaded with Coenzyme Q10 (CoQ10) (CoQ10@TNPs) for treatment of cardiac graft cold I/R injury in a murine heterotopic cardiac transplant model. The hybrid nanoparticles, composed of CaCO_3_/CaP/biotinylated carboxymethylchitosan (CaCO_3_/CaP/BCMC), were synthesized through the coprecipitation method. To obtain CoQ10@TNPs, we incorporated the mitochondria-targeting tetrapeptide SS31 onto the surface of the hybrid nanoparticles using biotin-avidin interactions. The SS31 peptide effectively targets the inner mitochondrial membrane by directly interacting with cardiolipin, regardless of the presence of mitochondrial membrane potential [6]. This effect is a unique benefit for delivering drugs directly to mitochondria in ischaemic cardiomyocytes under circumstances of the loss of mitochondrial membrane potential (MMP) in cold I/R-injured cardiomyocytes. Therefore, SS31 serves as an innovative peptide specifically directed towards mitochondria, enabling the targeted delivery of drugs to accumulate in the mitochondria of cold I/R-injured cardiomyocytes. Furthermore, the biocompatibility and biodegradability of the constituents in our hybrid vector, BCMC, CaCO_3_, and CaP, are excellent [9, 10].

In this study, we successfully synthesized CoQ10@TNPs and performed a series of studies to demonstrate the preclinical benefits of CoQ10@TNPs on cold I/R injury in HT. Initially, we conducted an analysis to determine the size distribution, morphology, surface potential, and biocompatibility of CoQ10@TNPs. Then, we explored the uptake and intracellular distribution of CoQ10@TNPs in H9c2 cells and DH. Finally, we verified that CoQ10@TNPs could efficiently deliver CoQ10 to the mitochondria of myocardial cells with slow and sustained release, thus protecting cells against oxidative injury and apoptosis. Our results demonstrated that CoQ10@TNPs effectively protect cardiomyocytes during cold storage in vitro. We also verified that CoQ10@TNP perfusion into the DH during cold storage protected against oxidative injury and apoptosis and significantly improved heart graft function.

## Methods and materials

### Main reagents and antibodies

BCMC (Additional file 1: Fig. S1A, B) and biotinylated SS31 (BSS31) (TP-WY-5088) (Additional file 1: Fig. S2A, B) were obtained from Ruixi Biotechnology (Xi’an, China). Streptavidin (9013-20-1) and CoQ10 (303-98-0) were obtained from Aladdin (Shanghai, China). Sodium phosphate dibasic dodecahydrate (Na_2_HPO_4_·12H_2_O) (10,039-32-4), anhydrous sodium carbonate (Na_2_CO_3_) (497-19-8), and calcium chloride (CaCl_2_) (10,043-52-4) were obtained from Sinopharm Chemical Reagent Co., Ltd. (Shanghai, China). IR780 iodide (IR780) (207,399-07-3) was purchased from Sigma-Aldrich (Shanghai, China). Anti-Bcl2 (26,593-1-AP), anti-cytochrome c (10,993-1-AP), anti-Bax (50,599-2-Ig), anti-COXIV (11,242-1-AP), anti-ERK (11,257-1-AP), anti-p-ERK (28,733-1-AP), anti-JNK (24,164-1-AP), anti-p-JNK (80,024-1-RR), anti-MAPK (14,064-1-AP), and anti-p-MAPK (28,796-1-AP) antibodies were obtained from Proteintech (Wuhan, China). Anti-8-OHdG (bs-1278R) antibodies were obtained from Bioss (Beijing, China). Anti-CD11b (ab133357) antibodies were obtained from Abcam, and anti-Ly6G (551,459) antibodies were obtained from BD Biosciences. Anti-β-actin (GB15003-100) antibodies were obtained from Servicebio (Wuhan, China). Fluorochrome-conjugated antibodies for mouse FITC-CD4 (F2100401), PE-CD8α (F21008A02), and PE-CD11c (F21011C02) were purchased from MultiSciences Biotechnology (Hangzhou, China). The TdT-mediated dUTP nick end-labelling (TUNEL) assay kit (C1089), dihydroethidium (S0063), CCK-8 assay kit (C0037), Annexin V-FITC/PI apoptosis detection kit (C1062S), JC-1 assay kit (C2003S), MPTP assay kit (C2009S), tissue mitochondria isolation kit (C3606), cell mitochondria isolation kit (C3601), and MitoTracker Green (C1048) were obtained from Beyotime Biotechnology (Shanghai, China). MitoSOX (40778ES50) was acquired from Yisheng Biotechnology (Shanghai, China). A mitochondrial DNA isolation kit (ab65321) and protein carbonyl content assay kit (ab126287) were acquired from Abcam (Shanghai, China). A cardiac troponin I (cTnI) enzyme-linked immunosorbent assay (ELISA) kit (CTNI-1-HS) was acquired from Life Diagnostics, Inc. (PA, USA). The creatine kinase MB isoenzyme (CK-MB) assay kit (H197-1–1), lactate dehydrogenase (LDH) assay kit (A020-2–2), malondialdehyde (MDA) assay kit (A003-1–2), superoxide dismutase (SOD) assay kit (A001-3–2), reduced glutathione (GSH) assay kit (A006-2–1), alanine aminotransferase (ALT) assay kit (C009-2–1), aspartate aminotransferase (AST) assay kit (C010-2–1), urea assay kit (C013-1–1), and creatinine (CREA) assay kit (C011-2–1) were from Nanjing Jiancheng Biotechnology (Nanjing, China). Mouse immunoglobulin G (IgG) (EK271) and IgM ELISA kits (EK276) were purchased from MultiSciences Biotechnology (Hangzhou, China). Mouse complement C4 (C4) (E-EL-M3020) and C3 ELISA kits (E-EL-M0330c) were from Elabscience (Wuhan, China).

### Preparation and characterization of CoQ10@TNPs

CoQ10@TNPs were successfully synthesized as we described earlier with slight modifications [11]. In brief, a mixture of 0.0106 g of Na_2_CO_3_ and 0.0358 g of Na_2_HPO_4_·12H_2_O was dissolved in 10 ml of ultrapure water. Subsequently, 20 μl of a CoQ10 solution (2 mg/ml) was introduced and thoroughly mixed to obtain solution B. Then, a mixture of 0.010 g of BCMC and 0.022 g of CaCl_2_ was dissolved in 10 ml of ultrapure water to obtain solution A. Afterwards, solution B was added dropwise to solution A under stirring at 50 °C for 30 min to synthesize biotinylated nanoparticles. Next, the solution underwent centrifugation at a speed of 10,000 rpm for a duration of 30 min, and the liquid portion above the sediment was removed. Then, the biotinylated nanoparticles were suspended in 2 ml of ultrapure water, and 15 mg of streptavidin was added while stirring at 37 °C for a duration of 30 min. Subsequently, the streptavidin-conjugated nanoparticle solution underwent centrifugation at a speed of 10,000 rpm for a duration of 30 min. Afterwards, streptavidin-conjugated nanoparticles were redissolved in 2 ml of ultrapure water, and then, 0.1 µg of BSS31 was added while stirring at 37 °C for a duration of 30 min to synthesize CoQ10@CaCO_3_/CaP/BCMC/streptavidin/BSS31 targeting nanoparticles named CoQ10@TNPs. For nontargeting nanoparticles (CoQ10@NPs), the synthetic steps were the same as for CoQ10@TNPs, except that the surface was not modified by BSS31. Next, the CoQ10@TNP solution was placed in a cuvette and then exposed to a Zetasizer (Nano ZS, Malvern Instruments) to measure its particle size and surface potential. Transmission electron microscopy (TEM) (JEOL-100CXII, Japan) was employed to examine the morphology of CoQ10@TNPs. The drug-loaded nanoparticles were centrifuged to collect the supernatant, and the entrapment efficiency of the drug-loaded nanoparticles was determined. The drug release characteristics of CoQ10@TNPs were assessed with the dialysis technique. The concentration of CoQ10 was determined using high-performance liquid chromatography (HPLC) (Agilent 1100).

### Cell culture

H9c2 cells were obtained from the Shanghai Institutes of Biological Sciences and grown in DMEM with 10% FBS and 1% penicillin‒streptomycin and maintained under standard conditions (5% CO_2_, 95% O_2_, 37 °C).

### Cytotoxicity of CoQ10@TNPs in H9c2 cells

H9c2 cells were cultured for 24 h and incubated with CoQ10@TNPs at different time points (0 h, 24 h, 48 h, and 72 h) and concentrations (0 µM, 5 µM, 10 µM, and 20 µM). Then, a CCK-8 assay kit was applied to assess cell viability. The cell apoptosis rate was also evaluated by using flow cytometry (FCM) (CytoFlex, Beckman Coulter).

### In vivo toxicity of CoQ10@TNPs in mice

Mice were separately treated with 200 μl of PBS or CoQ10@TNPs by intravenous (i.v.) injection. After administration, the weight of the mice was recorded daily, and their overall behaviour was monitored daily for any indications or manifestations of illness. After one week, mouse plasma samples were obtained, and the levels of AST, ALT, CREA, and urea were measured with AST, ALT, CREA, and urea detection kits. Moreover, the weights of the hearts, livers, spleens, lungs, and kidneys were measured. HE staining was applied to observe the tissue morphology.

The tissue distribution of CoQ10@TNPs was also examined in C57BL/6 mice. In this case, major organs were collected at predefined time points after i.v. injection of IR780-labelled CoQ10@TNPs, followed by in vivo organ imaging with an In Vivo Imaging System (IVIS Spectrum, PerkinElmer, U.S.A.).

### Haematological compatibility of CoQ10@TNPs in mice

Six- to eight-week-old male C57BL/6 mice were separately treated with 200 μl of PBS or CoQ10@TNPs. After one week, mouse blood samples were obtained to measure the parameters red blood cells (RBCs), white blood cells (WBCs), and blood platelets (PLTs) using standard haematological tests. For haemolysis evaluation of CoQ10@TNPs in vitro, fresh blood was collected and then incubated with PBS (negative control), CoQ10@TNPs, or pure water (positive control) at 25 °C for 2 h.

### Immunogenicity of CoQ10@TNPs in mice

Six- to eight-week-old male C57BL/6 mice were treated with 50 μl of PBS or CoQ10@TNPs by i.v. injection once a day five times. After 6 days, mouse blood samples were obtained to measure the levels of IgG, IgM, C4, and C3. Moreover, spleens were collected for quantification of the percentage of CD11c^+^ cells, CD4^+^ T cells, and CD8^+^ T cells using FCM.

### Cellular uptake of CoQ10@TNPs

H9c2 cells were seeded in 6-well plates. After 24 h, 2 ml of UW solution containing IR780-labelled CoQ10@TNPs was added to each well, and the cells were grown in a hypoxic environment (0.5% O_2_, 5% CO_2_, 94.5% N_2_, 4 °C) for 0 h, 0.5 h, 1 h, and 2 h. Then, the cells were fixed with 4% paraformaldehyde and subsequently stained with DAPI. A fluorescence microscope was applied to acquire the fluorescence images, and FCM was used to detect the fluorescence intensity.

### Cellular localization of CoQ10@TNPs

H9c2 cells were treated with 2 ml of UW solution containing IR780-labelled CoQ10@NPs or CoQ10@TNPs for 2 h. Then, the cells were treated with MitoTracker Green for 30 min. Finally, the distribution of IR780-labelled CoQ10@NPs or CoQ10@TNPs in the cells was observed by a laser scanning confocal microscope (LSCM).

### DH uptake of CoQ10@TNPs

The DHs were infused with one millilitre of chilled heparin through the inferior vena cava (IVC). Subsequently, cold UW solution containing IR780-labelled CoQ10@NPs or CoQ10@TNPs was infused into the DHs from the aorta. The DHs were subsequently removed and stored in a precooled UW solution containing IR780-labelled CoQ10@NPs or CoQ10@TNPs at 4 °C for 12 h. After PBS washes, the DHs were observed by an in vivo imaging system.

### Determination of the CoQ10 concentration in DH tissue and mitochondria

For the measurement of CoQ10 tissue concentration, the indicated tissue-specific isolation buffer was used to homogenize the DHs after briefly rinsing them in saline to eliminate CoQ10 from the surface of the organ. The mixture was subjected to centrifugation at 700 ×*g* for 5 min at a temperature of 4 °C to separate intact cells and debris. A portion of the resulting liquid was extracted to measure the amount of CoQ10. The mitochondria of DHs were isolated using a Tissue Mitochondria Isolation Kit according to the manufacturer’s instructions. Tissue CoQ10 was extracted following the steps described in detail previously [12]. The concentration of CoQ10 was determined with HPLC.

### An in vitro cold I/R injury model

A preexisting hypoxia-reoxygenation model was employed to replicate in vivo cold I/R injury [13]. In short, H9c2 cells were treated with 2 ml of UW solution containing PBS, SS31, CoQ10, CoQ10@NPs, TNPs, or CoQ10@TNPs under a hypoxic environment (0.5% O_2_, 5% CO_2_, 94.5% N_2_, 4 °C) for 12 h. After 12 h of hypoxia, the UW solution was substituted with regular cell culture medium, and cells were grown in a standard environment (5% CO_2_, 95% O_2_, 37 °C) for 24 h to simulate the reperfusion stage.

### Determination of MPTP opening

The MPTP Assay Kit was utilized for the detection of MPTP channel activation. After different interventions, H9c2 cells were treated with 1 ml of calcein AM solution (1 ×) with 10 μl of CoCl_2_ solution (100 ×) for 30 min at 37 °C in the dark. Afterwards, the calcein AM solution was replaced with fresh preheated culture medium and incubated at 37 °C for 30 min in the dark. Finally, the cells were resuspended in 400 μl of buffer for FCM analysis.

### Measurement of intracellular ROS

Heart grafts were made into frozen sections and subjected to staining with 5 μM DHE. H9c2 cells were also subjected to incubation with 5 μM DHE. The graft heart sections were imaged utilizing a fluorescence microscope, and the ROS level in H9c2 cells was assessed by FCM analysis.

### Measurement of mtROS

Living cells were incubated with MitoSOX Red and MitoTracker Green for 20 min at 37 °C in darkness. Then, the cells were washed with Hanks balanced salt solution and analysed by LSCM.

### Determination of MMP

The JC-1 Assay Kit was utilized following the provided guidelines to identify alterations in MMP. After different interventions, the H9c2 cells were subjected to incubation with JC-1 staining working solution at 37 °C for 20 min in darkness. Fluorescence microscopy was applied to determine the alterations in MMP.

### Cell apoptosis analysis

After different interventions, the H9c2 cells were collected and resuspended in 100 μl of binding buffer. Afterwards, the cells were stained with 5 μl of Annexin V-FITC and PI staining solution. Finally, the cells were resuspended in 400 μl of binding buffer and analysed by FCM.

### Murine cervical heterotopic HT

As previously mentioned, a syngeneic recipient underwent heterotypic cardiac transplantation following the established procedure [14]. DHs were procured using a predominantly conventional method, albeit with a minor alteration involving the insertion of small tubes (measuring 5 × 1 mm) into the IVC and aorta beneath the heart. The DHs were infused with one millilitre of chilled heparin through the IVC. Subsequently, cold UW solution containing PBS, SS31, CoQ10, CoQ10@NPs, TNPs, or CoQ10@TNPs was infused into the DHs from the aorta. The DHs were subsequently removed and stored in a precooled UW solution containing PBS, SS31, CoQ10, CoQ10@NPs, TNPs, or CoQ10@TNPs at 4 °C for 12 h. Prior to the recipient anastomosis, the DHs underwent flushing using a fresh UW solution that contained PBS, SS31, CoQ10, CoQ10@NPs, TNPs, or CoQ10@TNPs. The DHs were implanted in the neck region of the recipient mice using a cuff technique, where the donor aorta and pulmonary artery were connected to the recipient carotid artery and external jugular vein, respectively, by joining their ends together.

### Echocardiography and haemodynamics

Graft function of the heart was evaluated using a high-frequency ultrasound scanning system (Vevo 2100, Toronto, Canada). Briefly, the mice were initially administered anaesthetic induction utilizing a mixture of 4% isoflurane and oxygen. The hair in the cervical region of the mouse was completely eliminated using a depilatory cream. The positioning of the cardiac transplant was assessed through manual examination. Vevo Analysis software was utilized to compute the left ventricular ejection fraction (LVEF) and fractional shortening (LVFS).

### HE staining

Heart grafts were harvested from mice on day 1 post-transplantation and then fixed in 4% formalin. The paraffin-embedded sections underwent deparaffinization using xylene, followed by rehydration using a series of ethanol concentrations, leading to immersion in PBS, before analysis of the tissue morphology through HE staining.

### Biochemical analysis in serum

CK and LDH detection kits and cTnI ELISA kits were applied to measure the content of CK, LDH, and cTnI in recipient mouse serum in accordance with the guidelines provided by the manufacturer.

### Analysis of the cellular antioxidant system

The SOD Assay Kit, MDA Assay Kit, and GSH Assay Kit were applied to measure the activity of SOD and the contents of MDA and GSH in the grafts in accordance with the guidelines provided by the manufacturer.

### Immunofluorescence staining

The graft sections first underwent standard dehydration and antigen retrieval procedures. Next, the graft sections were treated with QuickBlock™ blocking buffer for 15 min. Then, the graft sections were incubated with anti-CD11b antibodies, anti-Ly6G antibodies, and anti-8-OHdG antibodies at 4 °C for 14 h. Afterwards, the graft sections were treated with Cy3-labelled secondary antibodies and incubated at room temperature for 1 h, after which DAPI was used as a counterstain for the nuclei. A fluorescence microscope was applied to capture the images.

### Oxidative damage to mtDNA and protein carbonyl content assays

A commercial kit from Abcam was utilized to extract mtDNA, and the assessment of mtDNA damage was conducted using qPCR [15]. The primer sequences were as follows: the long and short strand shared forward primers, F_c_: 5′-GCCAGCCTGACCCATAGCCATAAT; reverse primer of long strands, R_l_: 5′-GAGAGATTTATGGGTGTAATCGG; reverse primer of short strands, R_s_: 5′-GCCGGCTGCGTATCTACGTTA. The PCR system and parameters were established in accordance with the methodology described in a previous investigation [16]. The protein carbonyl content of samples from specified groups was determined using the Abcam protein carbonyl content assay kit, following the guidelines provided by the manufacturer.

### Examination of mitochondrial morphology in heart grafts

The graft tissues of mice were fixed with glutaraldehyde for 48 h. Then, the tissue was exposed to osmic acid for a duration of 4 h prior to being embedded in epoxy resin. An ultrathin sample was prepared, and the structure of mitochondria was observed through TEM.

### TUNEL staining

The graft sections first underwent dewaxing with xylene and then were exposed to proteinase K solution in the absence of DNase and supplemented with 0.3% Triton X-100 to enhance tissue permeability. Next, the TUNEL assay reagents were employed for DNA break labelling at 37 °C. Subsequently, PBS was used to wash the samples, followed by the application of DAPI to label the cell nuclei. Finally, fluorescence microscopy was applied to capture the images.

### Real-time quantitative PCR (RT‒qPCR) analysis

The expression levels of target genes (IL-6, MCP-1, and TNF-α) were calculated using RT‒qPCR. The specific primers used here were as follows: IL-6 forward: CAGAAGGAGTGGCTAAGGACCA, reverse: ACGCACTAGGTTTGCCGAGTAG; TNF-α forward: ATGAGAAGTTCCCAAAT GGC, reverse: CTCCACTTGGTGGTTTGCTA; MCP-1 forward: GAAGGAATG GGTCCAGACAT, reverse: ACGGGTCAACTTCACATTCA; β-actin forward: GGCTGTATTCCCCTCCATCG, reverse: CCAGTTGGTAACAATGCCATGT. The 2^–ΔΔCt^ method was applied to calculate the gene expression levels.

### Western blotting analysis

Western blotting analysis was used to determine the relative expression of total protein or mitochondrial protein. The antibodies used here were as follows: cleaved caspase-3, Bcl2, cytochrome c, Bax, COXIV, ERK, JNK, MAPK, p-ERK, p-JNK, p-MAPK, and β-actin. The bands were visualized with the ChemiDoc™ XRS + system (Bio-Rad Laboratories, Inc., USA).

### Data statistics and analysis

The mean ± standard deviation (SD) values were used to present the data, and GraphPad Prism 9.0 software was utilized for statistical analysis. Once the data were evaluated for normal distribution and equal variance, comparisons between two groups were conducted using Student’s *t* test. Furthermore, for multiple comparisons, a one-way ANOVA was performed, followed by Tukey’s post hoc test. If the data did not pass these tests, the Mann‒Whitney test was employed to compare the two groups. A p value < 0.05 was considered statistically significant. Each experiment was conducted a minimum of three times.

## Results

### Characterization of CoQ10@TNPs

**Fig. 1:**
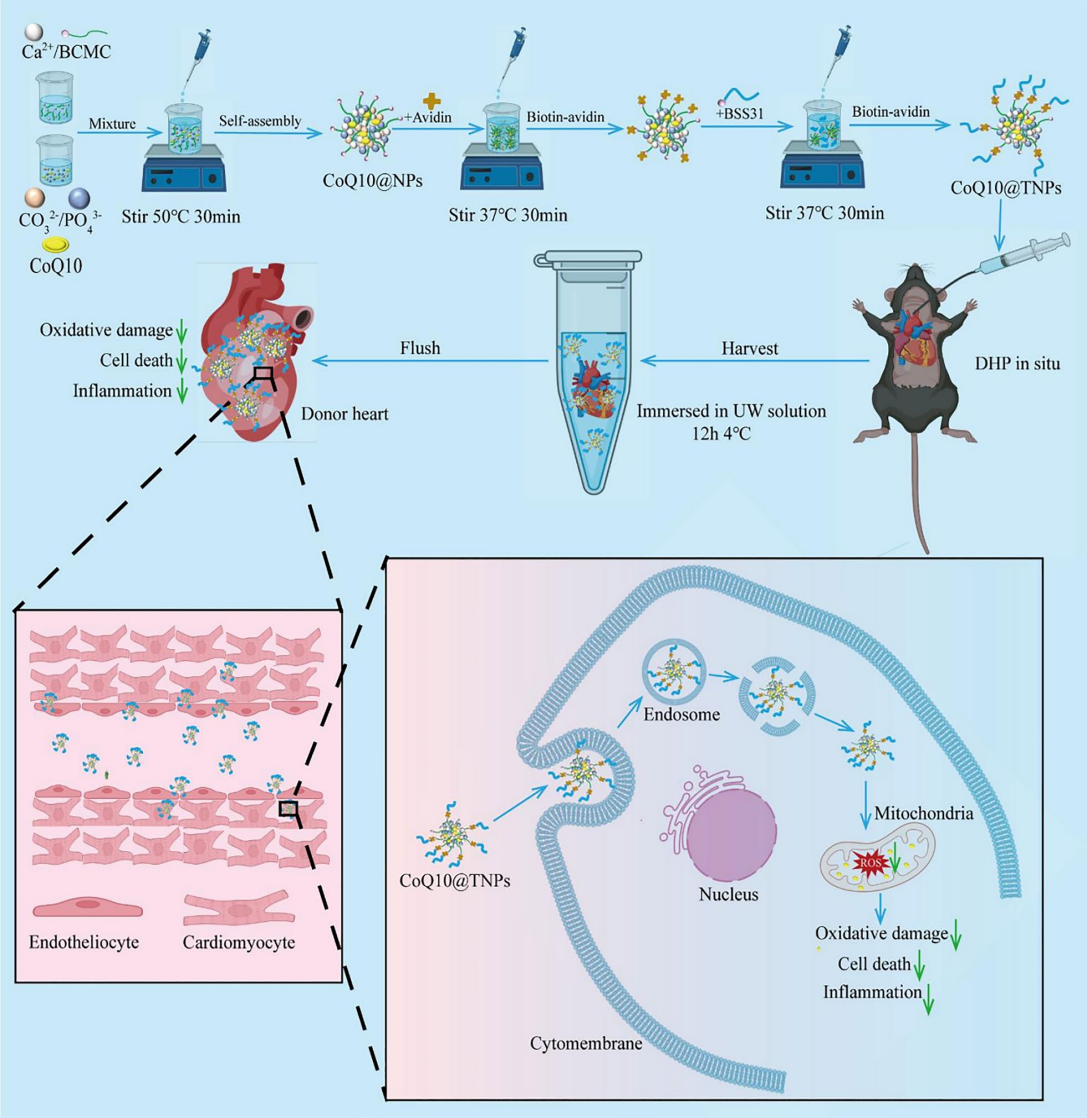
Schematic diagram of the formation and mechanism of the therapeutic effects of CoQ10@TNPs in cold I/R injury of HT

**Fig. 2:**
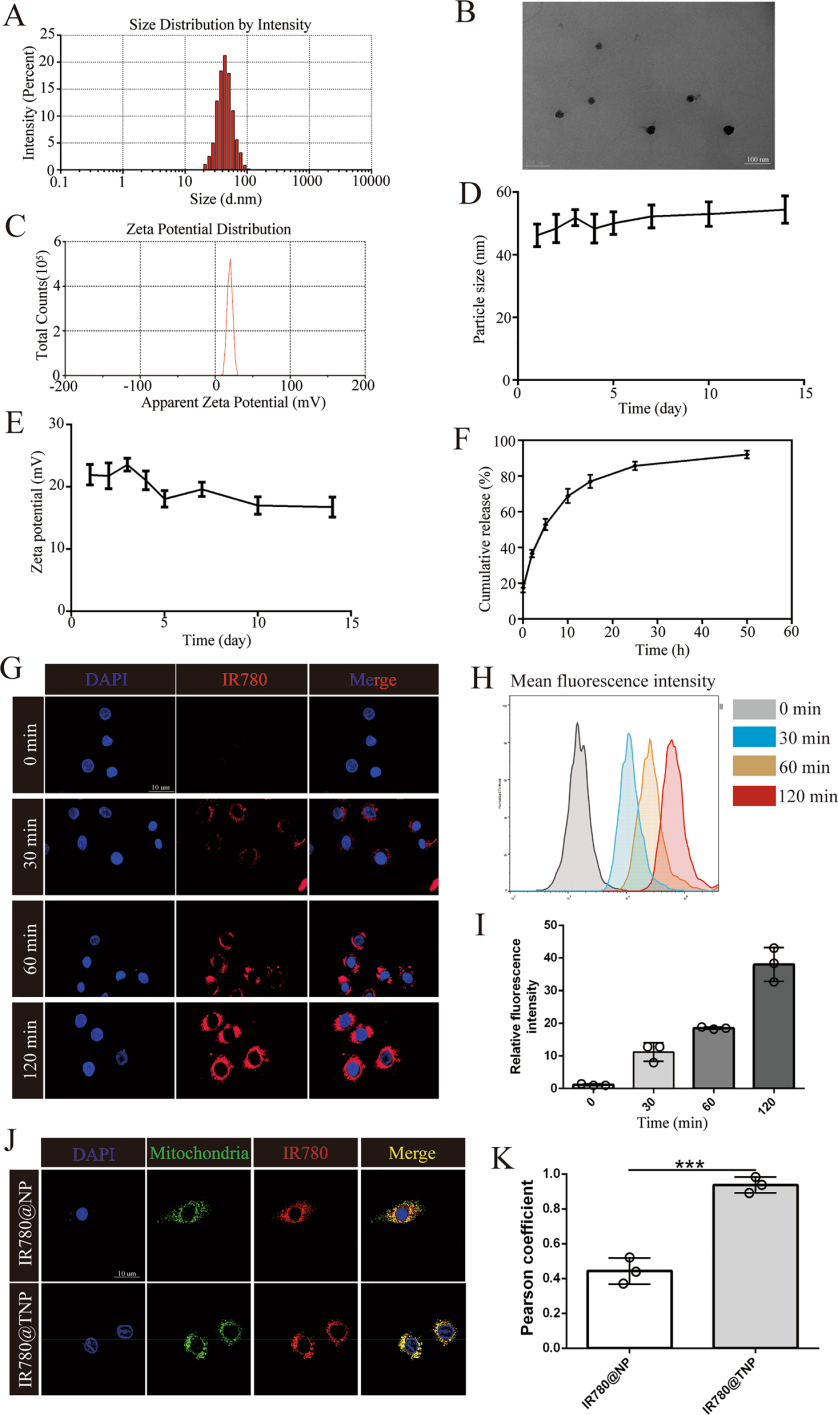
Characterization and intracellular trafficking of CoQ10@TNPs. **A** Diameter distribution of CoQ10@TNPs. **B** TEM morphology of CoQ10@TNPs. **C** Surface potential of CoQ10@TNPs. **D** The particle size of CoQ10@TNPs in ultrapure water. **E** The zeta potential of CoQ10@TNPs in ultrapure water. (F) CoQ10 release kinetics of CoQ10@TNPs. **G** Fluorescence microscopy images showing time-dependent cellular uptake of CoQ10@TNPs in H9c2 cells. **H**, **I** FCM analysis of the cellular uptake of CoQ10@TNPs in H9c2 cells after incubation for different periods. **J**, **K** Colocalization of mitochondria and CoQ10@TNPs in H9c2 cells. Data are represented as the mean ± SD (n = 3). ns: no significance; ***p < 0.001

The hybrid nanoparticles, composed of CoQ10@CaCO_3_/CaP/BCMC, were synthesized through the coprecipitation method. The mitochondrial targeting peptide SS31 was added on the surface of CoQ10@CaCO_3_/CaP/BCMC nanoparticles through the utilization of the biotin/avidin interaction, which is known as one of the most robust noncovalent interactions found in nature [17, 18], to obtain CoQ10@CaCO_3_/CaP/BCMC/streptavidin/BSS31 targeting nanoparticles termed CoQ10@TNPs (Fig. 1). The encapsulation efficiency of CoQ10 was determined to be 53.2 ± 2.7%, resulting in a dosage of 21.3 μg of CoQ10 per dose. Then, to verify the successful construction of CoQ10@TNPs, we measured the diameter, surface potential, and morphology using a Zetasizer and TEM. As demonstrated in Fig. 2A–C, the mean size of the CoQ10@TNPs was 45.7 nm in diameter. Since BSS31 is on the surface of CoQ10@TNPs, the surface potential of CoQ10@TNPs was + 19.56 mV. The TEM images revealed that the nanoparticles exhibited a spherical morphology and were evenly dispersed, devoid of any clustering or aggregation.

### Stability and CoQ10 release kinetics of CoQ10@TNPs

The stability of CoQ10@TNPs in ultrapure water is shown in Fig. 2D, E. The findings demonstrated that CoQ10@TNPs remained stable for a period exceeding 15 days. This outcome occurred due to the repulsive force generated by the positively charged nanoparticles. The stability began to decline after a period of 15 days, possibly indicating the destruction of CoQ10@TNPs. To elucidate the drug release characteristics of CoQ10@TNPs, we used a release medium consisting of 40% ethanol. The CoQ10 release kinetics of CoQ10@TNPs are shown in Fig. 2F, and CoQ10 was released gradually from the nanoparticles, achieving 60% accumulative release after 7 days.

### Uptake of CoQ10@TNPs by H9c2 cells and intracellular distribution

CoQ10@TNPs are anticipated to perform their biological function in H9c2 cells. Hence, we first investigated the cellular internalization patterns of CoQ10@TNPs in H9c2 cells under cold temperature conditions (4 °C). Time-dependent uptake of CoQ10@TNPs in H9c2 cells was observed through fluorescence microscopy and FCM. In addition, we found that many nanoparticles accumulated in the cells at 2 h (Fig. 2G–I). Moreover, we examined the intracellular distribution of CoQ10@TNPs. As shown in Fig. 2J, K, after H9c2 cells were treated with IR780-labelled CoQ10@TNPs for 2 h, the merged CLSM images showed the overlap between IR780 red fluorescence and MitoTracker Green fluorescence. This finding indicated that a large amount of IR780 was delivered into mitochondria. In contrast, after H9c2 cells were cultured with IR780-labelled CoQ10@NPs, the merged images displayed a slightly yellow colour, which indicated that a small amount of IR780 was delivered into mitochondria. Based on these data, we concluded that CoQ10@TNPs could be absorbed by H9c2 cells and localized to mitochondria.

### Biosafety evaluation of CoQ10@TNPs in vitro and in vivo

Prior to conducting in vitro and in vivo biological assessments and therapeutic investigations, we initially analysed the safety profiles of CoQ10@TNPs. The incubation of H9c2 cells with CoQ10@TNPs at a concentration of 5 μM for different times or for 24 h at different concentrations resulted in a significant preservation of cell viability, as indicated by the cytotoxicity tests (Additional file 1: Fig. S3A, B). Furthermore, CoQ10@TNPs had no effect on H9c2 cell apoptosis (Additional file 1: Fig. S3C–F). Then, the toxicity of CoQ10@TNPs was also evaluated in mice. To investigate the localization of CoQ10@TNPs in various organs of mice, we initially administered IR780-labelled CoQ10@TNPs into mice (50 mg/kg) through tail vein injection (Additional file 1: Fig. S4A). Organ imaging showed that CoQ10@TNPs were mainly distributed in lung tissue, followed by the liver, heart, spleen, and kidney (Additional file 1: Fig. S4B). All mice exhibited normal daily behaviours and maintained their body weight after receiving a single i.v. injection of CoQ10@TNPs, with no instances of mortality (Additional file 1: Fig. S4C). On the seventh day following treatment, there were no notable changes in the organ index of major organs, such as the heart, liver, spleen, lung, and kidney, in the CoQ10@TNP groups compared to the PBS group (Additional file 1: Fig. S4D). No significant alterations were observed in the levels of typical biomarkers associated with hepatic and renal functions, such as ALT, AST, urea, and CREA, compared with those of the group treated with PBS (Additional file 1: Fig. S4E–H). Similarly, examination of HE-stained pathological sections of vital organs demonstrated no detectable cellular necrosis or tissue damage following administration of CoQ10@TNP treatment (Additional file 1: Fig. S4I). In addition, we evaluated the blood compatibility of CoQ10@TNPs. In vitro experiments indicated that the haemolysis of mouse erythrocytes was minimally affected by CoQ10@TNPs (Additional file 1: Fig. S5A). Similarly, mice treated with CoQ10@TNPs showed normal levels of haematological parameters, including WBCs, RBCs, and PLTs (Additional file 1: Fig. S5B–D). Based on these data, we concluded that CoQ10@TNPs demonstrated favourable safety profiles.

### Immunogenicity of CoQ10@TNPs in mice

Since CoQ10@TNPs include BCMC, BSS31, and PEG-2000, which may cause an immune response, we observed characteristic immunological parameters, including IgG, IgM, C4, and C3, in the peripheral blood of mice following repeated administrations of CoQ10@TNPs (Additional file 1: Fig. S6A). Repeated administrations of CoQ10@TNPs were observed to have no significant impact on the levels of IgG, IgM, C4, and C3, indicating that there were no abnormal increases in these immune markers (Additional file 1: Fig. S6B–E). We also collected the mouse spleen, which is one of the most important immune organs in mice, for quantification of the percentage of CD11c^+^ cells and CD4^+^ and CD8^+^ T cells by FCM. The results showed that CoQ10@TNP treatments did not cause statistically significant changes in the percentage of CD11c^+^ cells, CD4^+^ T cells, or CD8^+^ T cells (Additional file 1: Fig. S6F–J). These preliminary results indicated that CoQ10@TNPs did not induce notable immune reactions.

### CoQ10@TNPs ameliorated oxidative stress and mitochondrial injury in cold I/R-injured H9c2 cells

**Fig. 3:**
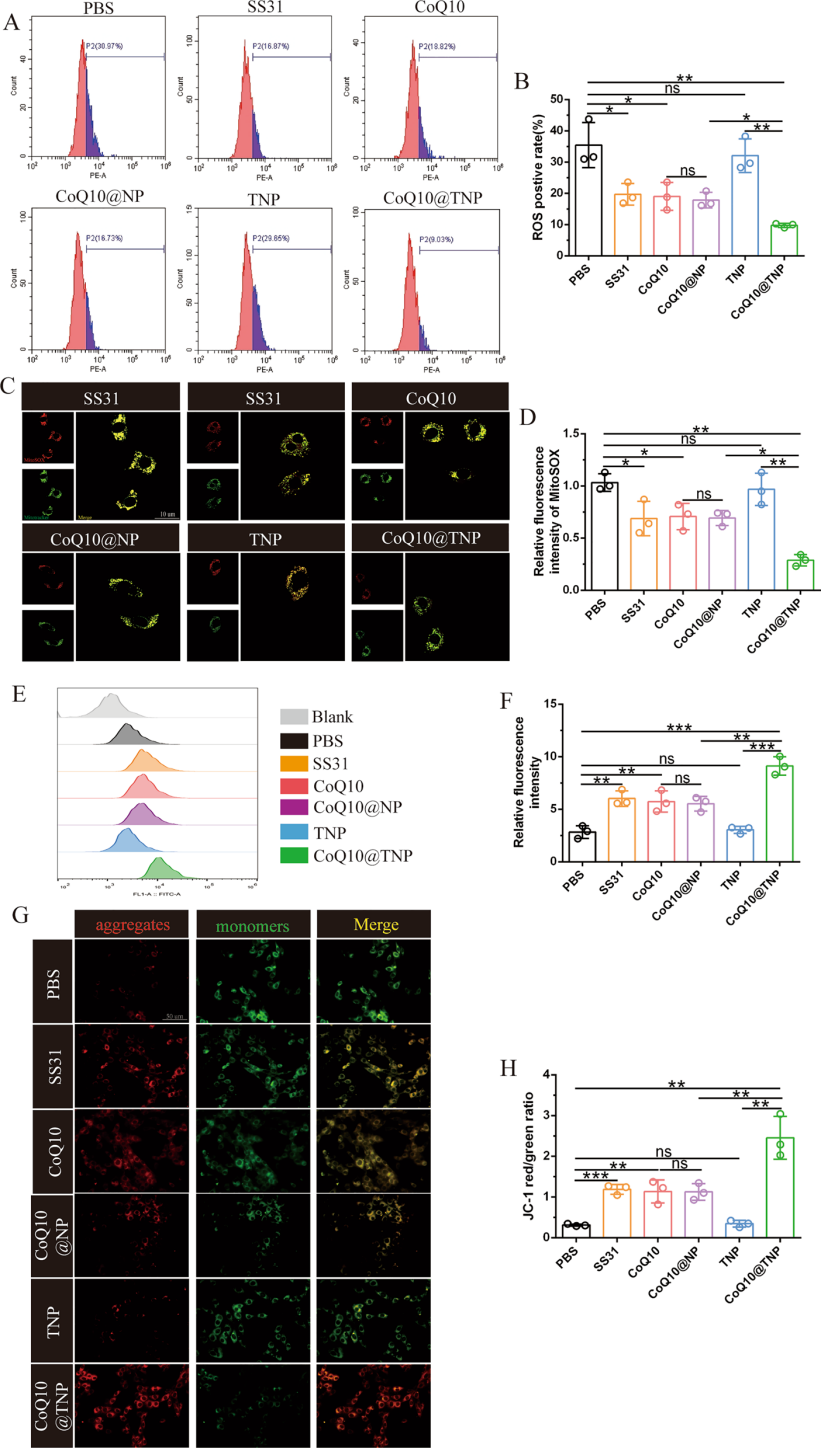
CoQ10@TNPs ameliorated oxidative stress and mitochondrial injury in cold I/R-injured H9c2 cells. **A**, **B** ROS content in H9c2 cells measured by DHE staining. **C**, **D** mtROS content in H9c2 cells measured by MitoSOX staining. **E**, **F** Fluorescence intensity of calcein AM in H9c2 cells measured by FCM. **G**, **H** MMP was measured by JC-1 staining. Data are represented as the mean ± SD (n = 3). ns: no significance; *p < 0.05; **p < 0.01; ***p < 0.001

During cold I/R injury, the main pathological event is the excessive generation of ROS. Therefore, the generation of ROS was initially investigated using FCM. We discovered that cold I/R injury increased intracellular ROS levels, while SS31, CoQ10, CoQ10@NPs, and CoQ10@TNPs efficiently inhibited this process. Furthermore, we found that CoQ10@TNPs exhibited the most potent suppression of ROS accumulation in cold I/R injury H9c2 cells (Fig. 3A, B). Since mitochondria are the major source of ROS production, we further carried out research on ROS accumulation in mitochondria by MitoSOX staining. As expected, compared to those in the PBS and TNP groups, the accumulation of ROS in mitochondria of H9c2 cells treated with SS31, CoQ10, and CoQ10@NPs was modestly reduced. In contrast, CoQ10@TNPs substantially reduced ROS accumulation in mitochondria (Fig. 3C, D). Collectively, these data suggested that CoQ10@TNPs played an important role in reducing intracellular ROS production in cold I/R-injured H9c2 cells.

The excessive generation of ROS has been documented to worsen mitochondrial impairment, leading to cellular death. Moreover, considering the above data demonstrating that CoQ10@TNPs could decrease intracellular ROS production, we explored whether CoQ10@TNPs could improve mitochondrial function in cold I/R-injured H9c2 cells. Hence, we first assessed MPTP opening in cold I/R-injured H9c2 cells using the MPTP Assay Kit. The effect of CoQ10@TNPs on MPTP channel activation in cold I/R-injured H9c2 cells is shown in Fig. 3E, F. The decrease in fluorescence intensity observed in cold I/R-injured H9c2 cells suggested an elevated occurrence of MPTP opening in cold I/R-injured H9c2 cells. When cold I/R-injured H9c2 cells received prior treatment using SS31, CoQ10, CoQ10@NPs, and CoQ10@TNPs, they showed more intense fluorescence. This finding indicated that MPTP channel activation was restrained by SS31, CoQ10, CoQ10@NPs, and CoQ10@TNPs. In addition, the results indicated that CoQ10@TNPs exhibited the most potent suppression of MPTP channel activation in cold I/R-injured H9c2 cells. The occurrence of MPTP opening within the inner mitochondrial membrane leads to a decline in the MMP. The alterations in MMP are illustrated in Fig. 3G, H. The ratio of red fluorescence intensity to green fluorescence intensity was decreased in cold I/R-injured H9c2 cells. However, after pretreatment with SS31, CoQ10, CoQ10@NPs, and CoQ10@TNPs, the fluorescence ratio of red to green was enhanced, which indicated a recovery of MMP. Moreover, CoQ10@TNPs resulted in a notable enhancement in MMP compared to SS31, CoQ10, and CoQ10@NPs. Altogether, these results revealed that CoQ10@TNPs could deliver CoQ10 into the mitochondria, which consequently mitigated mitochondrial injury in H9c2 cells during prolonged cold storage.

### CoQ10@TNPs reduced H9c2 cell apoptosis during cold I/R injury in vitro

**Fig. 4:**
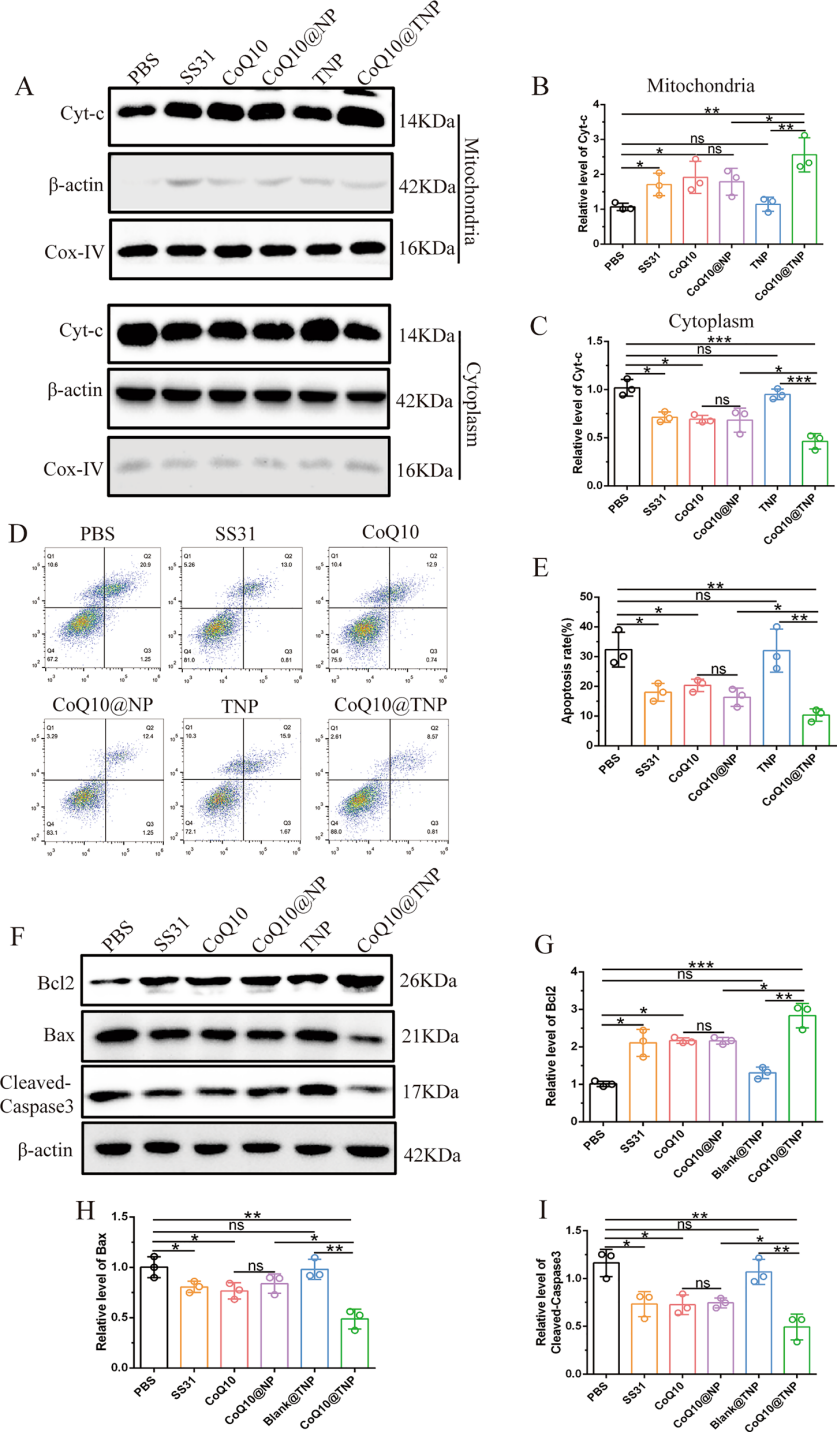
CoQ10@TNPs reduced H9c2 cell apoptosis during cold I/R injury in vitro. **A**–**C** The level of cytochrome c in the cytoplasm and mitochondria. **D**, **E** The apoptosis rate of H9c2 cells was evaluated by FCM. **F**–**I** Western blotting was employed to evaluate the protein levels of Bax, Bcl-2, and C-caspase-3. Data are represented as the mean ± SD (n = 3). ns: no significance; *p < 0.05; **p < 0.01; ***p < 0.001

Apoptosis is an important mechanism involved in programmed cell death and plays a crucial role in various pathophysiological conditions, particularly during cold I/R injury. Generally, there are two signalling pathways that regulate apoptosis: one pathway that is mediated by cell death receptors and another pathway that is mediated by mitochondria. Many studies have shown that prolonged cold storage can decrease mitochondrial function, thus causing a mitochondria-dependent apoptotic pathway [3, 19]. Therefore, we next investigated whether CoQ10@TNPs could alleviate mitochondria-dependent apoptosis in H9c2 cells induced by cold I/R injury. We initially measured the amount of cytochrome c in mitochondria and cytoplasm. The results showed that cold I/R injury promoted the translocation of cytochrome c from the mitochondria to the cytoplasm, while SS31, CoQ10, CoQ10@NPs, and CoQ10@TNPs efficiently inhibited cytochrome c release. Moreover, compared with SS31, CoQ10, and CoQ10@NPs, CoQ10@TNPs had a stronger inhibitory effect (Fig. 4A–C). After being released into the cytoplasm, cytochrome c triggers the activation of caspase-3, which is an acknowledged intrinsic cell apoptotic pathway via mitochondria. Therefore, we next performed Annexin V-FITC/PI double staining to examine the impact of different treatments on the apoptosis of H9c2 cells. The results showed that SS31, CoQ10, CoQ10@NPs, and CoQ10@TNPs reduced the cardiomyocyte apoptosis triggered by cold I/R injury. Additionally, CoQ10@TNPs markedly decreased the percentage of apoptosis in cold I/R-injured H9c2 cells (Fig. 4D, E). Western blotting analysis was conducted to evaluate the levels of apoptosis-related proteins. Consistent with the above results, CoQ10@TNPs dramatically increased the inhibitor of the apoptosis protein Bcl2 and restrained the expression of the proapoptotic proteins cleaved caspase-3 (C-caspase-3) and Bax in cold I/R-injured H9c2 cells (Fig. 4F–I). Growing evidence demonstrates that the mitogen-activated protein kinase (MAPK) signalling pathway plays important roles in cardiomyocyte apoptosis during myocardial I/R [20]. Therefore, Western blotting was performed to investigate the activation of MAPK pathways during cold I/R progression. The results showed that SS31, CoQ10, CoQ10@NPs, and CoQ10@TNPs reduced the activation of p38, JNK, and ERK. Additionally, CoQ10@TNPs markedly decreased the activation of MAPK pathways (Additional file 1: Fig. S7A, B). On account of the above data, we confirmed that CoQ10@TNPs could significantly decrease the apoptosis of H9c2 cells damaged by cold I/R.

### CoQ10@TNPs accumulated in donor cardiac tissue at 4 °C

**Fig. 5:**
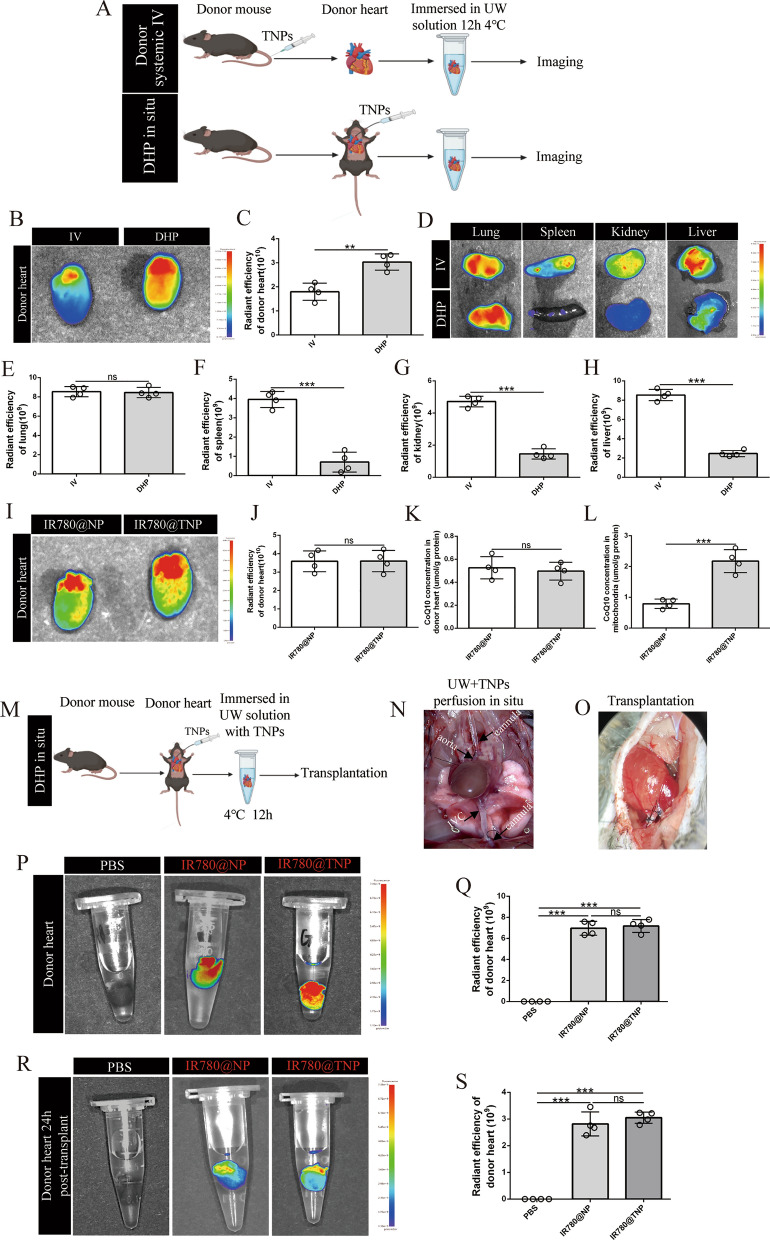
DH delivery, kinetics, and trafficking of CoQ10@TNPs. **A** The schematic describes two different ways to deliver CoQ10@TNPs to DH. **B**, **C** The content of CoQ10@TNPs in the DH of the two groups was determined by organ in vivo imaging. **D**–**H** The content of CoQ10@TNPs in the lung, liver, spleen, and kidney of the two groups was determined by organ in vivo imaging. **I**, **J** The content of CoQ10@NPs and CoQ10@TNPs in DH was determined by organ in vivo imaging. **K** The content of CoQ10 in DH tissues was evaluated by HPLC. **L** The content of CoQ10 in the mitochondria of DH was evaluated by HPLC. **M** Schematic of DH perfused with CoQ10@TNPs. **N** DH underwent flushing in situ with CoQ10@TNPs via a tiny tube (5 × 1 mm) in the ascending aorta and IVC. **O** The DH was implanted into the neck of the recipient. **P**, **Q** The content of CoQ10@TNPs in DH was determined by organ in vivo imaging before transplantation. **R**, **S** The content of CoQ10@TNPs in DH was determined by organ in vivo imaging 24 h after transplantation. Data are represented as the mean ± SD (n = 4). ns: no significance; *p < 0.05; **p < 0.01; ***p < 0.001

Given that CoQ10@TNPs protect H9c2 cells from cold I/R injury in vitro, we wanted to further evaluate the protective function of CoQ10@TNPs on the DH during cold preservation. Therefore, we first investigated how to most efficiently deliver CoQ10@TNPs into the DH during cold preservation. Drug loading into the DH may be most efficient when blood is perfused and beating is maintained. However, in a clinical environment, the systemic administration of CoQ10@TNPs would have an impact on every organ. Furthermore, achieving controlled delivery in an unstable or hypotensive donor is a major challenge. Fortunately, in the context of organ transplantation, a unique medical scenario arises where the organ is available outside the body, presenting a potential avenue for utilizing nanotechnology to administer therapeutics directly to the transplanted organ. Hence, we first measured the drug delivery efficiency of systemic i.v. injection (IV) 15 min before removing the heart and DH perfusion (DHP) in situ (Fig. 5A). We found that DHs from the DHP group exhibited a significantly stronger fluorescent signal than those from the IV group (Fig. 5B, C). Other organs, including kidneys, livers, and spleens, from the IV group showed more accumulation of CoQ10@TNPs and a higher MFI than that of the DHP group (Fig. 5D–H). These findings suggest that CoQ10@TNPs were successfully transported to the DH through DHP before organ transplantation and were effectively absorbed and localized within the heart without gathering in other organs.

Next, to evaluate the effectiveness of CoQ10@TNPs in delivering CoQ10 to the mitochondria of the DH, we performed DHP in situ with IR780-labelled CoQ10@TNPs or CoQ10@NPs (50 μmol/l) through a tube inserted into the ascending aorta. The DH was preserved in a cold solution with IR780-labelled CoQ10@TNPs or CoQ10@NPs, leading to additional absorption of IR780-labelled CoQ10@TNPs or CoQ10@NPs into the DH during cold storage. After 12 h of cold preservation, the DH was observed by an in vivo imaging system. The results showed that both CoQ10@TNPs and CoQ10@NPs can efficiently accumulate in DH tissue, and there is no difference in the amount of accumulation (Fig. 5I, J). To further confirm that both CoQ10@TNPs and CoQ10@NPs can efficiently accumulate in DH tissue, we measured the tissue CoQ10 concentration by HPLC. As expected, the concentration of CoQ10 in tissues did not differ between the two groups (Fig. 5K, I). Then, we isolated mitochondria from the DH and measured the CoQ10 concentration with HPLC. Mitochondria from the CoQ10@TNP flushed group revealed higher CoQ10 accumulation than mitochondria from the CoQ10@NP group (Fig. 5L). Collectively, these findings indicated that CoQ10@TNPs can efficiently transport CoQ10 to the mitochondria of the DH.

Since we previously clarified that CoQ10@TNPs can be efficiently loaded into the DH by DHP in situ, we wondered about the kinetics and trafficking of CoQ10@TNPs in the DH following transplantation into C57BL/6 mice. First, the DH was perfused with IR780-labelled CoQ10@TNPs, CoQ10@NPs, or PBS as described above. The DH was then harvested and immersed in IR780-labelled CoQ10@TNPs, CoQ10@NPs, or PBS with UW solution at 4 °C for 12 h (Fig. 5M–O), and images were captured with an in vivo imaging system. CoQ10@TNPs and CoQ10@NPs showed consistent distribution throughout the entire cardiac organ, indicating perfusion of heart tissue (Fig. 5P, Q). Next, the DH was implanted in the neck region of the C57BL/6 mouse using a cuff technique. The biodistribution of IR780-labelled CoQ10@TNPs and CoQ10@NPs was evaluated through bioluminescence analysis 24 h following HT using grafts obtained from the transplanted animals. Heart grafts perfused with IR780-labelled CoQ10@TNPs and CoQ10@NPs exhibited a fluorescent signal, contrasting with the absence of such a signal in heart grafts perfused with PBS (Fig. 5R, S). In addition, we found that the fluorescent signals of the IR780-labelled CoQ10@TNP- and CoQ10@NP-perfused groups after HT were the same, but the intensity was half of what it was before the transplantation. These data suggested that CoQ10@TNPs were successfully administered to the DH through perfusion before organ transplantation and maintained for more than 24 h after transplantation.

### CoQ10@TNPs reduced cold I/R injury and improved cardiac graft function

**Fig. 6:**
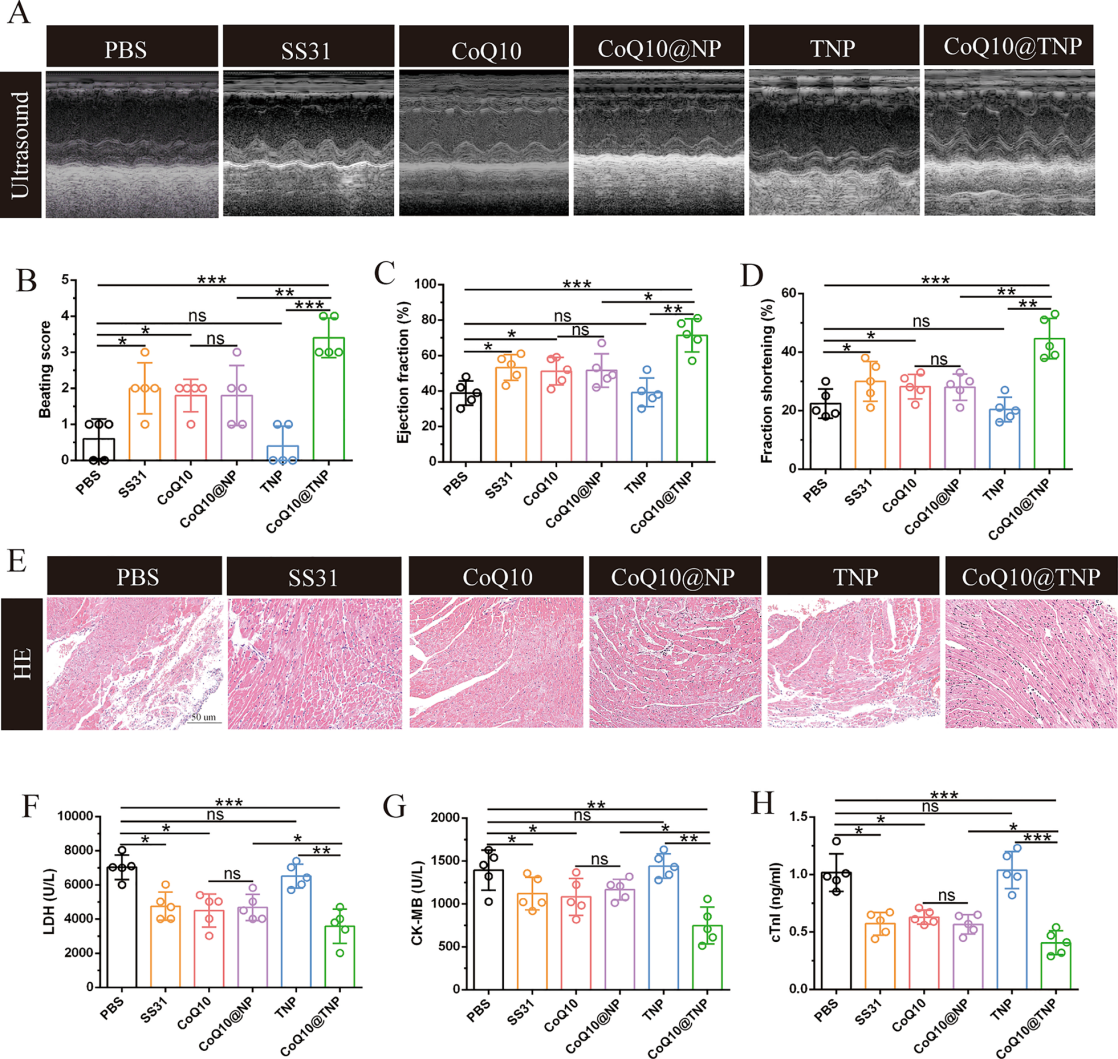
CoQ10@TNPs reduced cold I/R injury and improved cardiac graft function. **A** Function of the cardiac graft determined by ultrasound scanning on day 1 post-transplantation. **B** Beating score of the cardiac graft on day 1 post-transplantation. **C** Ejection fraction of the cardiac graft on day 1 post-transplantation. **D** Fractional shortening of the cardiac graft on day 1 post-transplantation. **E** HE staining of cardiac grafts on day 1 post-transplantation. **F**–**H** The serum content of LDH, cTnI, and CK-MB in the recipient. Data are represented as the mean ± SD (n = 5). ns: no significance; *p < 0.01; **p < 0.01; ***p < 0.001

Given that CoQ10@TNPs ameliorated cold I/R injury in vitro, we proposed to illustrate the in vivo efficacy of CoQ10@TNPs in a murine syngeneic heterotopic HT model of extended cold I/R injury. The dissected DH was perfused with cold UW solution containing PBS, SS31, CoQ10, CoQ10@NPs, TNPs, or CoQ10@TNPs and preserved in the UW solution containing PBS, SS31, CoQ10, CoQ10@NPs, TNPs, or CoQ10@TNPs at 4 °C for 12 h, and then, a syngeneic heterotopic HT procedure was performed. We discovered that DHs subjected to PBS and TNP treatment exhibited a weak palpable heartbeat, whereas the SS31-, CoQ10-, CoQ10@NP-, or CoQ10@TNP-treated grafts exhibited stronger palpable pulses. Moreover, the CoQ10@TNP-treated grafts exhibited the strongest palpable pulses. Next, to evaluate the function of heart grafts more objectively and accurately, we performed high-frequency ultrasonography on day 1 post-transplantation. We found that treatment with SS31, CoQ10, CoQ10@NPs, or CoQ10@TNPs augmented LVEF and LVFS in comparison with those of the PBS- and TNP-treated grafts. Furthermore, CoQ10@TNPs exhibited the most potent protective function in terms of LVEF and LVFS (Fig. 6A–D). This result indicated that CoQ10@TNPs obviously increased the function of the cold I/R-injured DH.

Since myocardial injury has an important impact on cardiac function, histopathological examination of the heart grafts was performed on day 1 post-transplantation. We first evaluated the cardiac tissue structure using HE staining. As expected, the heart grafts treated with PBS and TNPs exhibited significant necrosis and infarction in the myocardium, indicating extensive tissue damage. In contrast, the myocardium of the heart grafts subjected to SS31, CoQ10, CoQ10@NP, or CoQ10@TNP treatment exhibited a well-defined arrangement, with only localized regions of the grafts showing signs of hydropic degeneration injury. Moreover, the heart grafts treated with CoQ10@TNPs exhibited a more complete structure of the myocardium than those treated with SS31, CoQ10, or CoQ10@NPs (Fig. 6E). To further confirm the above results, we determined the levels of myocardial injury markers, including LDH, CK-MB, and cTnI, in serum. As shown in Fig. 6F–H, the contents of CK-MB, LDH, and cTnI were evidently decreased in the SS31, CoQ10, CoQ10@NP, and CoQ10@TNP groups. CoQ10@TNPs exhibited the most potent inhibitory impact on the levels of CK-MB, LDH, and cTnI. These data indicated that CoQ10 encapsulated in targeted nanoparticles can increase therapeutic efficacy and attenuate heart graft damage more effectively than SS31, CoQ10, TNPs, and CoQ10@NPs.

### CoQ10@TNPs attenuated cardiac graft mitochondrial oxidative damage

**Fig. 7:**
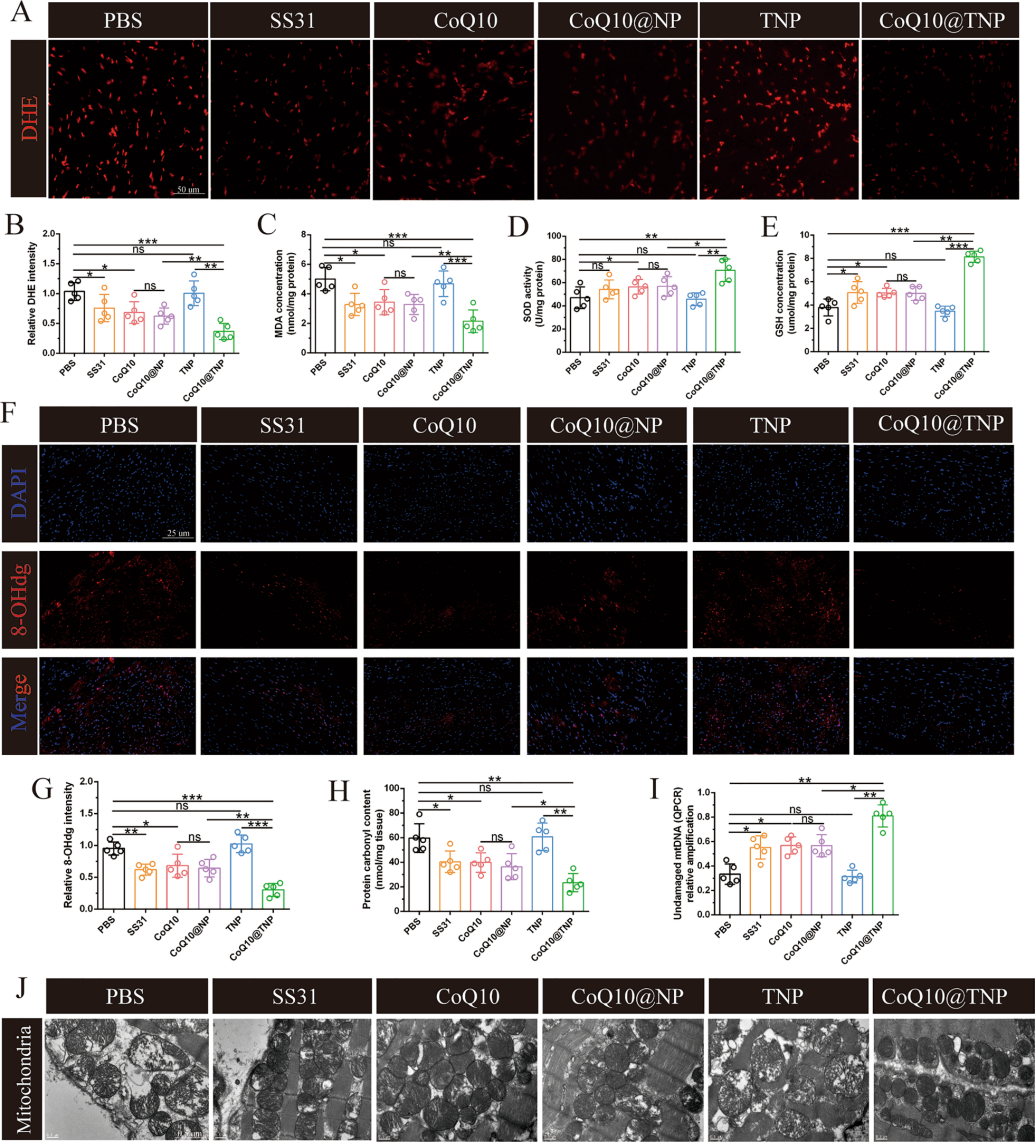
CoQ10@TNPs attenuated cardiac graft mitochondrial oxidative damage. **A**, **B** DHE staining was applied to evaluate the production of ROS in cardiac grafts. **C** The MDA level in cardiac grafts. **D** The activity of SOD in cardiac grafts. **E** The GSH level in cardiac grafts. **F**, **G** Immunofluorescence staining of 8-OHdg in cardiac grafts. **H** The level of protein carbonyl in cardiac grafts. **I** Undamaged mtDNA in the cardiac grafts. **J** Representative TEM images of mitochondria in cardiac grafts. Data are represented as the mean ± SD (n = 5). ns: no significance; *p < 0.05; **p < 0.01

Since oxidative stress is closely related to myocardial injury, we next conducted DHE staining to assess the level of cardiac graft ROS in mice. As shown in Fig. 7A, B, prolonged cold preservation significantly enhanced the generation of ROS in the heart graft, which was reduced by SS31, CoQ10, CoQ10@NP, or CoQ10@TNP administration. In addition, we found that CoQ10@TNPs exhibited a higher efficiency in preventing the formation of cardiac graft ROS than SS31, CoQ10, and CoQ10@NPs. To further make these experimental results more reliable, we measured the activity of SOD and the contents of MDA and GSH in cardiac grafts. The results showed that SS31, CoQ10, CoQ10@NP, or CoQ10@TNP treatment could significantly decrease the content of MDA and increase the activities of SOD and GSH in cardiac grafts. Of note, we found that the CoQ10@TNP-treated cardiac grafts had a lower content of MDA and higher activities of SOD and GSH than those treated with SS31, CoQ10, TNPs, or CoQ10@NPs (Fig. 7C–E). The results indicated that the administration of CoQ10@TNPs exerted a protective function against oxidative damage in vivo.

Mitochondrial oxidative damage is the main mechanism of oxidative stress-mediated myocardial injury. The protection of mitochondria against oxidative stress from ROS is therapeutically beneficial to the cold I/R injury of cardiac grafts, particularly in grafts subjected to extended cold preservation. Hence, we next investigated the protective effect of CoQ10@TNPs against mitochondrial oxidative injury. First, we determined the expression level of 8-OHdG, a biomarker that indicates mtDNA damage caused by ROS. The PBS and TNP groups exhibited the highest positive rates of 8-OHdG. We also found that the expression level of 8-OHdG was lower in the CoQ10@TNP group than in the SS31, CoQ10, and CoQ10@NP groups (Fig. 7F, G). To further assess the protection of CoQ10@TNPs against mitochondrial oxidative damage in prolonged cold-preserved cardiac grafts, we measured mtDNA damage by using qRT‒PCR and protein carbonyl generation (a biomarker of protein oxidative injury) with kits. As expected, similar results were obtained (Fig. 7H, I). Finally, we used TEM to capture the structure of mitochondria in cardiac grafts, and the results are shown in Fig. 7J. In the PBS and TNP groups, the mitochondria exhibited swelling, loss of cristae, and the presence of vacuoles. Nevertheless, the group treated with CoQ10@TNPs exhibited an increase in crista structures and a decrease in vacuolar shape. The aforementioned results suggested that CoQ10@TNPs effectively shielded mitochondria from oxidative harm induced by mtROS, thus preventing significant myocardial damage and graft dysfunction, particularly in grafts subjected to extended periods of cold storage.

### CoQ10@TNPs reduced cardiac graft apoptosis and inflammation

**Fig. 8:**
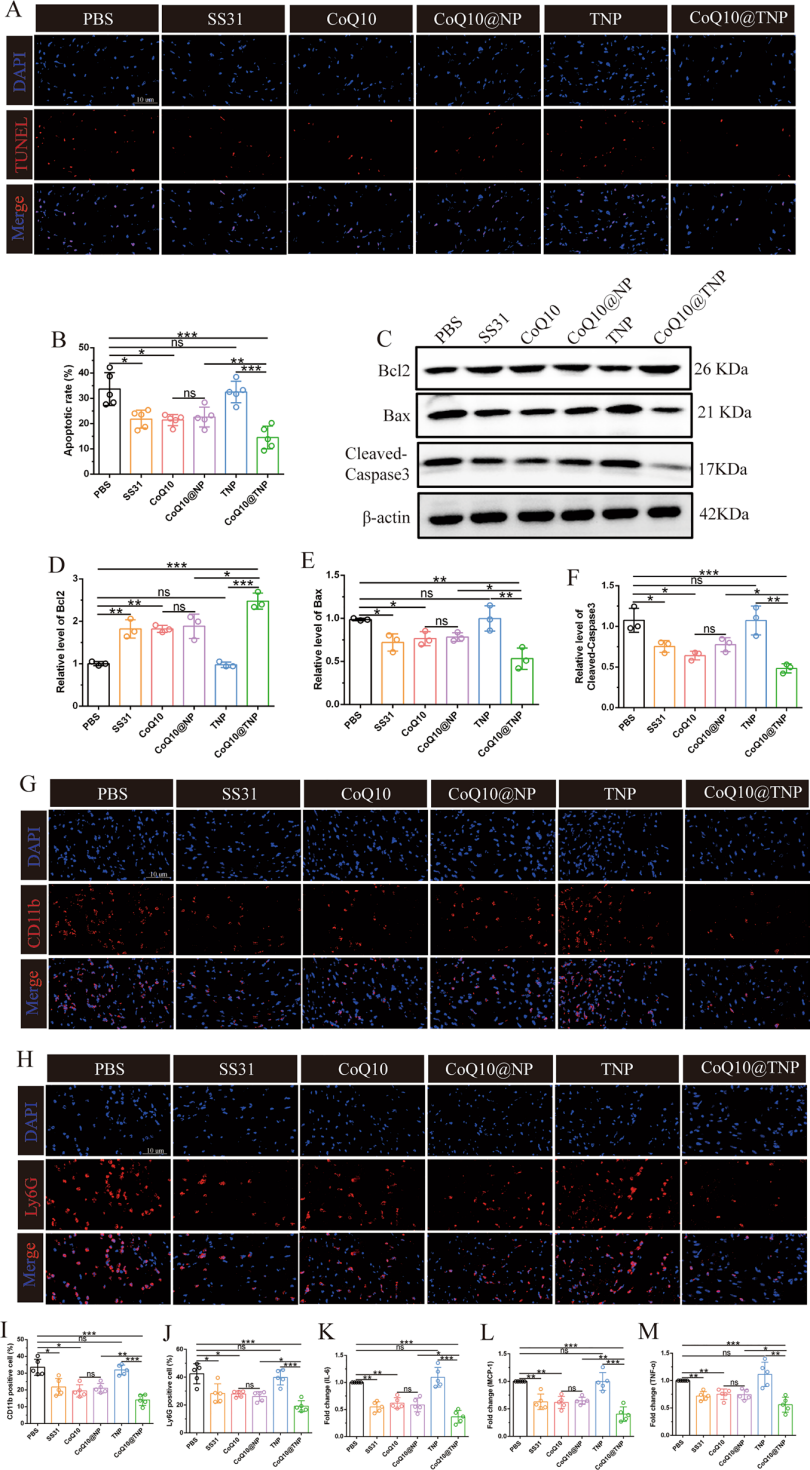
CoQ10@TNPs reduced apoptosis and inflammatory responses in cardiac grafts. **A**, **B** TUNEL staining is indicative of apoptotic cardiomyocytes in the transplanted heart. **C** Western blotting was employed to evaluate the protein levels of Bax, Bcl-2, and C-caspase-3. **D**–**F** Statistics of Bax, Bcl-2, and C-caspase-3 expression. **G**, **H** Immunofluorescence staining of CD11b and Ly6G in cardiac grafts. **I**, **J** Statistics of CD11b and Ly6G in cardiac grafts. **K**‒**M** The expression of IL-6, MCP-1, and TNF-α was estimated by qPCR in cardiac grafts. Data are represented as the mean ± SD (n = 5). ns: no significance; *p < 0.05; **p < 0.01; ***p < 0.001

Mitochondrial damage is one of the major mechanisms mediating apoptosis. Given that CoQ10@TNPs played a crucial role in mitigating myocardial apoptosis induced by cold I/R injury in vitro, we next performed an investigation to assess the potential of CoQ10@TNPs in safeguarding the heart graft against apoptosis induced by cold I/R injury in a murine syngeneic heterotopic HT model. Initially, TUNEL staining was conducted on the cardiac graft tissues of every group. In contrast to the PBS and TNP groups, the SS31, CoQ10, and CoQ10@NP groups had significantly lower apoptosis. As expected, the CoQ10@TNPs had a stronger inhibitory impact on apoptosis than SS31, CoQ10, and CoQ10@NPs (Fig. 8A, B). Furthermore, we performed Western blotting assays to analyse the protein expression levels of Bax, Bcl-2, and C-caspase-3 in the cardiac graft tissues of each experimental group. Cold I/R injury markedly upregulated Bax and C-caspase-3 expression, which was decreased in the SS31, CoQ10, and CoQ10@NP groups. Notably, CoQ10@TNPs had a stronger inhibitory impact on Bax and C-caspase-3 production. Nevertheless, Bcl-2 exhibited contrasting patterns among the groups (Fig. 8C–F). In conclusion, these aforementioned consequences indicated that CoQ10@TNPs effectively inhibits apoptosis during cold I/R injury of DH.

Since the release of damage-associated molecular patterns (DAMPs), such as HMGB1 and oxidized mtDNA, from oxidatively damaged cells induces recipient innate immune system activation via aseptic inflammation, which exacerbates damage to heart grafts, we next evaluated the role of CoQ10@TNPs in inducing recipient innate immune system activation in the HT model. At 24 h after transplantation, we measured the expression of CD11b and Ly6G by immunofluorescence to explore the effect of CoQ10@TNPs on macrophages and neutrophil infiltration in heart grafts. The results demonstrated that grafts in the PBS and TNP groups exhibited a higher rate of infiltration within the graft by CD11b-positive or Ly6G-positive cells than the SS31, CoQ10, CoQ10@NP, or CoQ10@TNP groups. Furthermore, compared with SS31, CoQ10, and CoQ10@NPs, CoQ10@TNPs were more effective in inhibiting the expression of CD11b and Ly6G (Fig. 8G–J). To conduct a more in-depth investigation into the function of CoQ10@TNPs in the inflammatory reaction of grafts that had experienced an extended period of cold storage, we also performed qRT‒PCR analysis to measure the mRNA expression of IL-6, MCP-1, and TNF-α in the heart grafts. As expected, the heart grafts from the CoQ10@TNP group exhibited prominent decreases in the mRNA expression of IL-6, MCP-1, and TNF-α compared with those from the PBS, SS31, CoQ10, TNP, and CoQ10@NP groups (Fig. 8K–M). Taken together, these data highlight the efficacy of CoQ10@TNPs in inhibiting the release of DAMPs, reducing the activation of the innate immune system, and mitigating damage to cardiomyocytes during cold I/R injury.

## Discussion

Organ transplantation is the optimal therapy for patients with terminal-stage organ failure. However, cold I/R injury, an inevitable occurrence during clinical transplantation, still results in higher morbidity and mortality due to initial graft dysfunction and restricts the potential to extend organ preservation periods and expand the pool of organ donors. Moreover, cold I/R injury can result in the release of DAMPs, including HMGB1 and mtDNA, which enhance the immunogenicity of the graft, thereby increasing the likelihood of immunological rejection [21]. Extended cold storage of human DHs is correlated with elevated cold I/R damage and a heightened incidence of early graft dysfunction following transplantation [22–25]. Therefore, developing more efficient preservation protocols to mitigate cold I/R injury to DH is critically important.

While the molecular mechanisms of cold I/R injury are not completely understood, a large body of studies has suggested that cold I/R injury is linked to excessive generation of ROS during the reestablishment of perfusion and simultaneous reintroduction of oxygen. The mitochondrion, as the primary organelle responsible for generating ROS, is critical in both oxidative metabolism and cellular apoptosis [26–28]. During cold storage and reperfusion, the progressive alteration of mitochondrial dysfunction contributes to the accumulation of ROS, which subsequently leads to additional mitochondrial dysfunction and cellular harm by triggering the opening of the MPTP. Additionally, high levels of ROS can lead to the oxidation and loss of function in lipids, proteins, and DNA, which can further contribute to the activation of the immune response, resulting in the generation of oxidative stress and causing damage to cells and tissues. In brief, mitochondrial dysfunction-induced oxidative stress, along with consequent systemic apoptosis and inflammation, plays a critical role in the development of cold I/R injury in grafts [5]. Hence, active targeted delivery of antioxidants to mitochondria provides a novel antioxidant therapy platform for preservation of the DH.

CoQ10, a vitamin-like substance that is soluble in fat, is primarily found within the inner membrane of mitochondria [29, 30]. Its primary function is to safeguard against the generation of harmful oxidants and the occurrence of detrimental modifications in DNA, proteins, and lipids [30, 31]. Several recent studies have provided evidence supporting the protective effects of CoQ10 against injury induced by I/R in various organs [32, 33]. Nonetheless, due to its strong hydrophobic properties and negative charge, CoQ10 faces restrictions in its ability to reach the intracellular mitochondria directly in its unassisted state. These mitochondria carry a substantial negative charge, further impeding the accessibility of CoQ10 [34]. Hence, the precise and efficient delivery of CoQ10 to mitochondria has emerged as a prominent area of research focus.

The advancements made in synthesizing and characterizing nanoscale materials have generated enthusiasm for improving the techniques for administering medications. The use of nanotechnology can enhance pharmacokinetic characteristics, resulting in the regulated and prolonged release of a substance and its targeted delivery. By incorporating targeting ligands, drug delivery systems can be designed to selectively transport drugs to specific sites, enhancing therapeutic efficacy while minimizing adverse effects [8]. Among the many drug delivery systems, natural polymer/inorganic nanocomposites have become a research hotspot for drug delivery due to their simple preparation, controllable particle size, high biological safety, and various properties [35]. Hence, based on previous research in our laboratory [11], we proposed a novel mitochondrion-targeted nanocarrier for delivering CoQ10, which was synthesized by self-assembly in an aqueous medium using BCMC, carbonate ions, and phosphate ions as raw materials.

CMC is an important water-soluble chitosan derivative that has a series of excellent functional properties, such as excellent biocompatibility and biodegradability, and has been widely used in the interdisciplinary fields of medicine and engineering. The chemical structure of CMC has two active groups, an amino group and a carboxyl group, and it easily combines with other macromolecules. More importantly, CMC is a polyanion and pH-sensitive material that can form controlled drug release hybrid nanoparticles by electrostatic interactions with metal cations, thereby delaying drug release, improving drug bioavailability, and enhancing the stability of inorganic nanoparticles [36]. Therefore, in our study, the CMC was biotinylated and then hybridized with calcium carbonate and calcium phosphate to form hybrid nanoparticles. In an aqueous solution, calcium ions with a positive charge interacted with the –COO– groups of CMCs as well as with negatively charged carbonate and phosphate ions, contributing to the assembly of CoQ10@CaCO_3_/CaP/BCMC nanoparticles.

Triphenylphosphine (TPP) is frequently employed as a functional group to direct the accumulation of nanoparticles within mitochondria. The function of TPP targeting is contingent upon the mitochondrial membrane potential [37]. Unfortunately, cold I/R injury leads to the loss of MMP. SS31 can penetrate the plasma membrane and specifically accumulate in the inner mitochondrial membrane of mitochondria by interacting with cardiolipin, independent of the mitochondrial membrane potential, and eliminate ROS [38, 39]. Furthermore, in contrast to TPP, SS31 does not induce mitochondrial depolarization even when present in millimolar concentrations, thus avoiding potential toxicity [6]. Therefore, SS31, as a mitochondria-targeting tetrapeptide, is added on the surface of the hybrid nanoparticles. Since SS31 was added on the surface of the nanoparticle, the surface potential of CoQ10@TNPs was + 19.6 mV. The positive charge not only enhances the ability of CoQ10@TNPs to cross the cell membrane but also enhances the biological stability of CoQ10@TNPs. However, we also found that CoQ10@TNPs could deliver more CoQ10 into the mitochondria of cold I/R-injured cardiomyocytes than CoQ10@NPs in vivo and in vitro. This finding indicated that SS31 plays a crucial role in maintaining the positive charge and targeted transport function of CoQ10@TNPs.

In brief, CoQ10@TNPs were mainly composed of BCMC, carbonate ions, phosphate ions and BSS31. BCMC and BSS31, which are common materials in targeting nanoparticle synthesis, have excellent biocompatibility. In addition, calcium phosphate and calcium carbonate are common substances in the human body and are nontoxic to biological systems. Consistent with this, our experimental results also showed that CoQ10@TNPs were safe, biocompatible, and biodegradable with no toxicity in vivo and in vitro. However, unlike the majority of models concerning systemic diseases, transplantation presents a distinctive situation where the affected organ can be directly targeted with therapeutic interventions through intraorgan delivery. Administration of targeted delivery of nanoparticles to the isolated heart has an advantage in that it avoids systemic absorption in the donor and recipient, thus minimizing safety concerns. In addition, by introducing an SS31-specific polypeptide to the delivery vector, the mitochondrion-targeted delivery system can induce mitochondria-specific antioxidant therapy. By efficiently eliminating mtROS, CoQ10@TNPs can reduce oxidative injury and inflammatory reactions in cold I/R-injured graft tissues and ultimately improve heart graft function. In the future, with the widespread applicability of marginal DH, the prevention of cold I/R injury will be the most decisive factor in improving heart graft function. Therefore, strategies that can effectively mitigate cold I/R injury will be particularly important. Since our mitochondrion-targeted delivery system exhibits dramatically enhanced antioxidant effects for preservation of the DH, the targeted drug delivery system we developed possesses good potential in mitigating cold I/R injury of the marginal DH. This study provides an efficient strategy for the preservation of DH.

## Conclusion

In summary, we developed a multifunctionalized CoQ10-loaded delivery system targeting mitochondria. Through self-assembly, CoQ10 could be effectively entrapped in the inner part of the delivery system, and SS31 could be added on the surface of the delivery system. Due to SS31, the delivery system can precisely deliver CoQ10 to the mitochondria of cold I/R-injured cardiomyocytes to effectively eliminate mtROS, thus reducing oxidative injury and inflammatory reactions in cold I/R-injured graft tissues and finally improving heart graft function. These discoveries indicate that therapeutic interventions aimed at reducing oxidative damage to mitochondria through targeted delivery systems could reduce the occurrence of post-transplant graft dysfunction. This method presents an opportunity to enhance existing results following HT and broaden the range of viable grafts for transplantation.

### Supplementary Information

**Additional file 1:** **Figure S1.** **A** The chemical structures of biotinylated carboxymethyl chitosan. **B** The ^1^HNMRspectrum analysis report of biotinylated carboxymethyl chitosan. **Figure S2.** **A** HPLC analysis report of biotinylated SS31. **B** Mass spectrometry analysis report of biotinylated SS31. **Figure S3.** **A**, **B** Cell viability of H9c2 cells following exposure to CoQ10@TNPs at various time and concentration intervals. **C**–**F** FCM was employed to investigate the apoptosis of H9c2 cells after treatment with CoQ10@TNPs for different time and concentration. Data are represented as mean ± SD (n = 3). ns: no significance. **Figure S4.** Toxicity evaluation of CoQ10@TNPs in mice. **A** Schematic diagram of experimental schemes. **B** CoQ10@TNPs distribution in the organs of the body. **C** Fluctuations in the body weight after i.v. injection of CoQ10@TNPs. **D** The major organs index after i.v. injection of CoQ10@TNPs. **E**–**H** Biochemical indicators associated with liver and kidney functions. **I** HE staining of typical major organs after i.v. injection of CoQ10@TNPs. Data are represented as mean ± SD (n = 4). ns: no significance. **Figure S5.** Blood compatibility of CoQ10@TNPs. **A** Hemolysis evaluation of CoQ10@TNPs in fresh erythrocytes. **B**–**D** The blood levels of WBC, RBC, and PLT after i.v. injection of CoQ10@TNPs. Data are represented as mean ± SD (n = 4). ns: no significance. **Figure S6.** The immunogenicity examination of CoQ10@TNPs. **A** Schematic diagram of experimental schemes. **B**, **C** The expression levels of IgM and IgG in blood samples after multiple administrations. **D**, **E** The expression levels of C3 and C4 in blood samples after multiple administrations. **F**, **H** The proportion of CD11c + immune cells after multiple administrations. **G**, **I**, **J** The proportion of CD4 + and CD8 + T cells after multiple administrations. Data are represented as mean ± SD (n = 4). ns: no significance. **Figure S7.** CoQ10@TNPs reduce the activation of MAPK pathway. **A** Western blotting was employed to evaluate the protein levels of P-ERK, ERK, P-JNK, JNK, P-p38, p38. **B**–**D** Statistical analysis of P-ERK, P-JNK, P-p38. Data are represented as mean ± SD (n = 3). ns: no significance; *p < 0.05; **p < 0.01.

## Data Availability

The datasets provided in this research are available in online repositories. The article or Supplementary Material contains the repository/repository's names and corresponding accession number(s).

## Abbreviations

HT: Heart transplantation
CoQ10: Coenzyme Q10
DH: Donor heart
TME: Transmission electron microscopy
DLS: Dynamic light scattering
CIT: Cold ischaemia time
ROS: Reactive oxygen species
MPTP: Mitochondrial permeability transition pore
mtDNA: Mitochondrial DNA
MMP: Mitochondrial membrane potential
BCMC: Biotinylated carboxymethylchitosan
BSS31: Biotinylated SS31
cTnI: Cardiac troponin I
CK-MB: Creatine kinase MB isoenzyme
LDH: Lactate dehydrogenase
MDA: Malondialdehyde
SOD: Superoxide dismutase
GSH: Reduced glutathione
ALT: Alanine aminotransferase
AST: Aspartate aminotransferase
HPLC: High-performance liquid chromatography
FCM: Flow cytometry
RBC: Red blood cell
WBC: White blood cell
IVC: Inferior vena cava
mtROS: Mitochondrial ROS
LSCM: Laser scanning confocal microscope
LVEF: Left ventricular ejection fraction
LVFS: Left ventricular fractional shortening
DAMP: Damage-associated molecular pattern
