# Supplementary material for: Mitochondrion-targeted carboxymethyl chitosan hybrid nanoparticles loaded with Coenzyme Q10 protect cardiac grafts against cold ischaemia‒reperfusion injury in heart transplantation
Source: J Transl Med. 2023 Dec 20;21:925. doi: 10.1186/s12967-023-04763-7 (PMC10734076; doi:10.1186/s12967-023-04763-7)
Supplement: Supplementary file 1 — Additional file 1: Figure S1. A The chemical structures of biotinylated carboxymethyl chitosan. B The 1HNMRspectrum analysis report of biotinylated carboxymethyl chitosan. Figure S2. A HPLC analysis report of biotinylated SS31. B Mass spectrometry analysis report of biotinylated SS31. Figure S3. A, B Cell viability of H9c2 cells following exposure to CoQ10@TNPs at various time and concentration intervals. C–F FCM was employed to investigate the apoptosis of H9c2 cells after treatment with CoQ10@TNPs for different time and concentration. Data are represented as mean ± SD (n = 3). ns: no significance. Figure S4. Toxicity evaluation of CoQ10@TNPs in mice. A Schematic diagram of experimental schemes. B CoQ10@TNPs distribution in the organs of the body. C Fluctuations in the body weight after i.v. injection of CoQ10@TNPs. D The major organs index after i.v. injection of CoQ10@TNPs. E–H Biochemical indicators associated with liver and kidney functions. I HE staining of typical major organs after i.v. injection of CoQ10@TNPs. Data are represented as mean ± SD (n = 4). ns: no significance. Figure S5. Blood compatibility of CoQ10@TNPs. A Hemolysis evaluation of CoQ10@TNPs in fresh erythrocytes. B–D The blood levels of WBC, RBC, and PLT after i.v. injection of CoQ10@TNPs. Data are represented as mean ± SD (n = 4). ns: no significance. Figure S6. The immunogenicity examination of CoQ10@TNPs. A Schematic diagram of experimental schemes. B, C The expression levels of IgM and IgG in blood samples after multiple administrations. D, E The expression levels of C3 and C4 in blood samples after multiple administrations. F, H The proportion of CD11c + immune cells after multiple administrations. G, I, J The proportion of CD4 + and CD8 + T cells after multiple administrations. Data are represented as mean ± SD (n = 4). ns: no significance. Figure S7. CoQ10@TNPs reduce the activation of MAPK pathway. A Western blotting was employed to evaluate the protein levels of P-ERK, ERK, P-JNK, JNK [file 12967_2023_4763_MOESM1_ESM.docx]

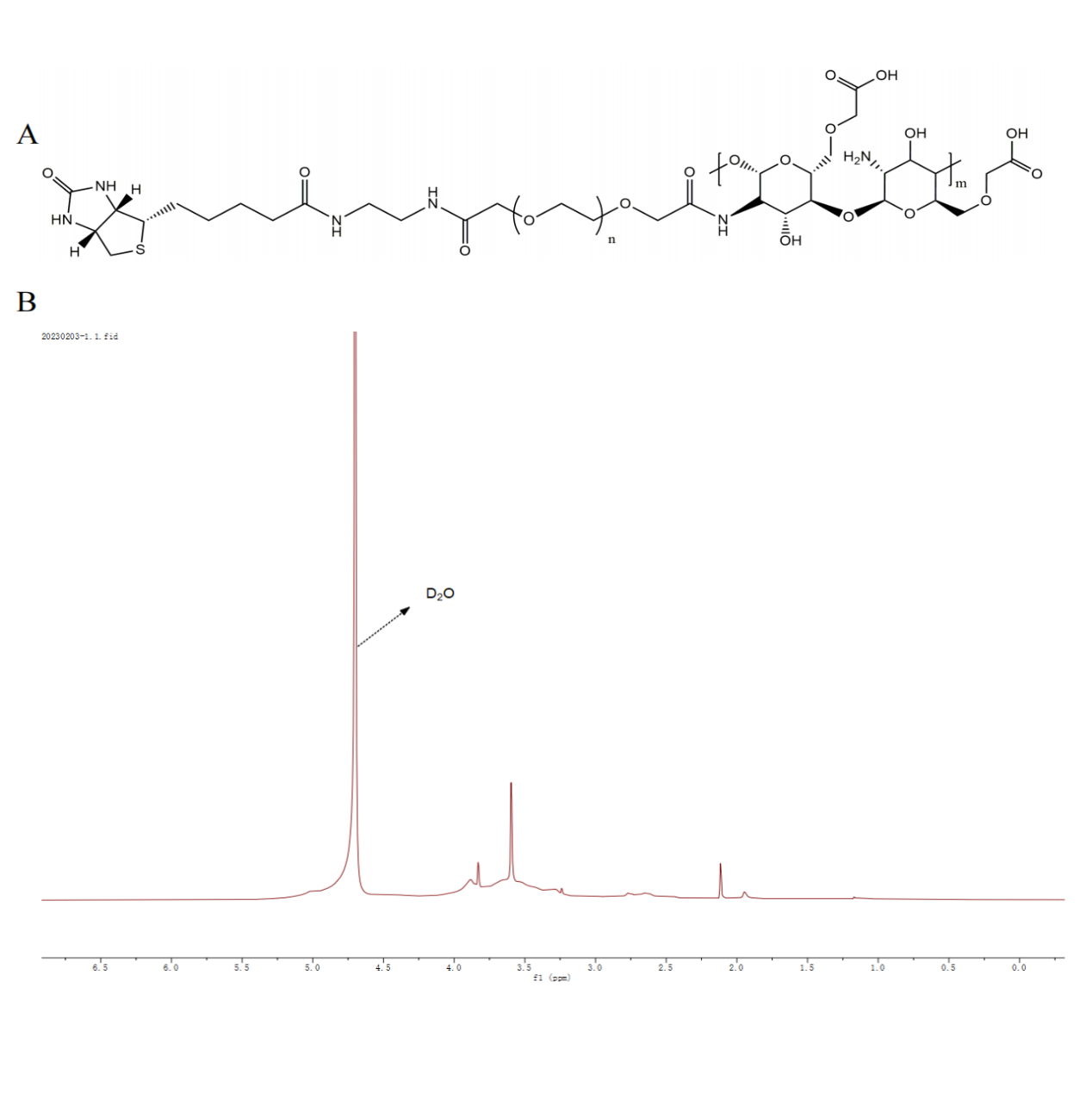


**Figure S1.** (A) The chemical structures of biotinylated carboxymethyl chitosan. (B) The ^1^HNMRspectrum analysis report of biotinylated carboxymethyl chitosan.

B

A


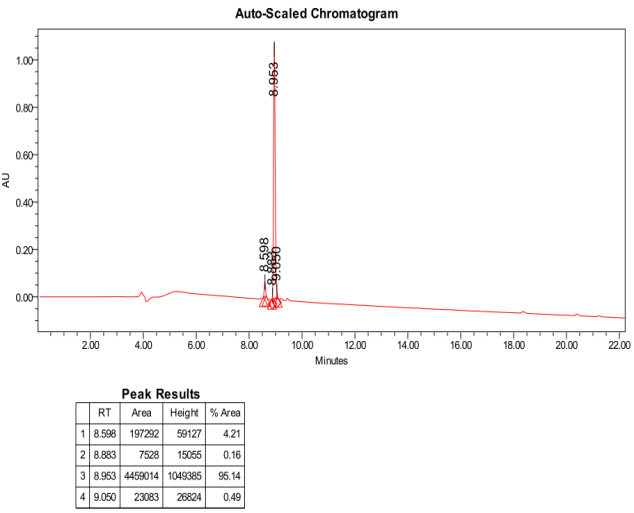

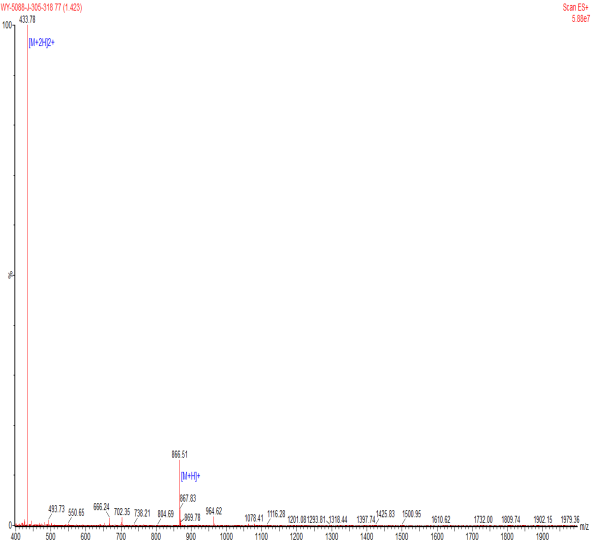


**Figure S2.** (A) HPLC analysis report of biotinylated SS31. (B) Mass spectrometry analysis report of biotinylated SS31.


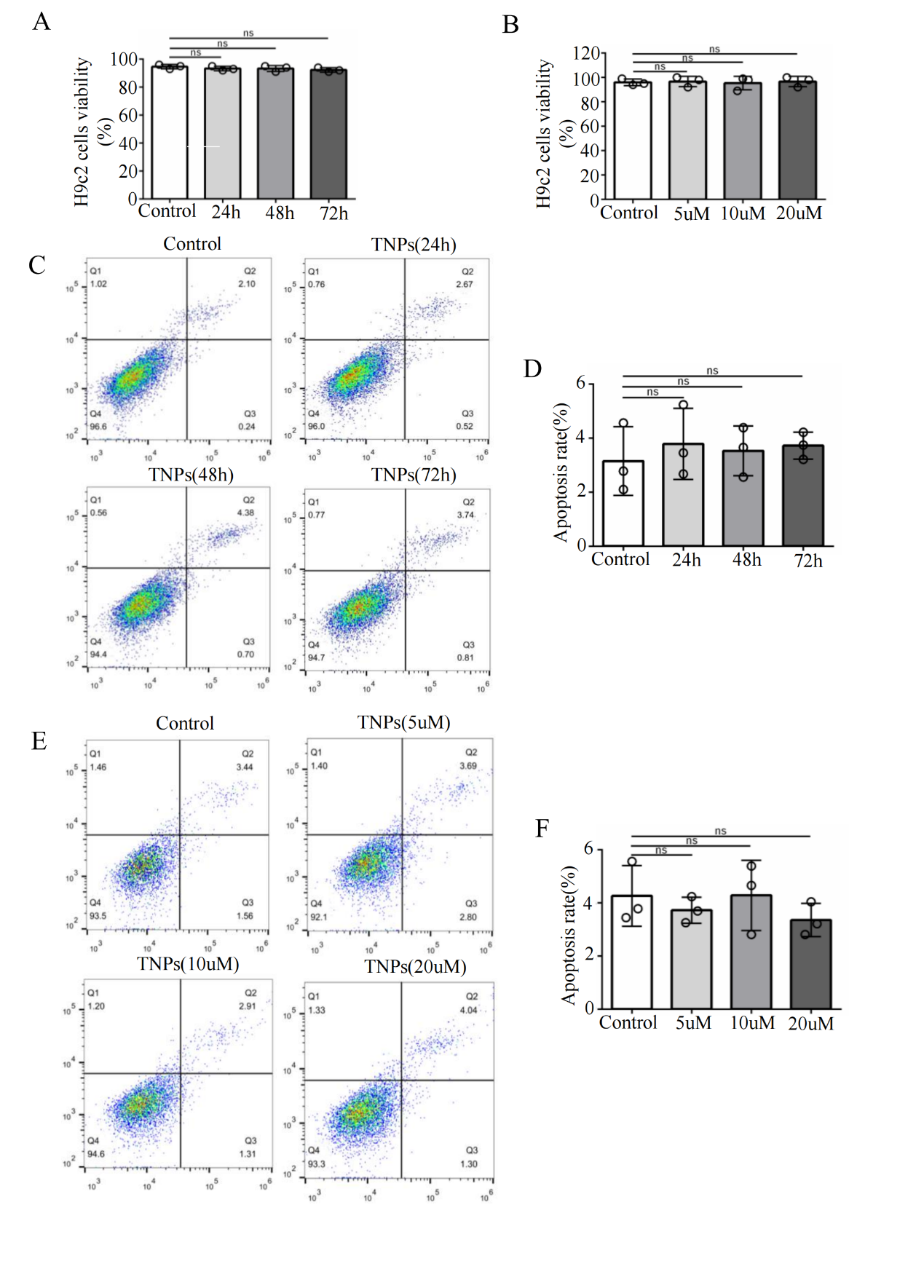
**Figure S3.** (A, B) Cell viability of H9c2 cells following exposure to CoQ10@TNPs at various time and concentration intervals. (C-F) FCM was employed to investigate the apoptosis of H9c2 cells after treatment with CoQ10@TNPs for different time and concentration. Data are represented as mean ± SD (n = 3). ns: no significance.

**
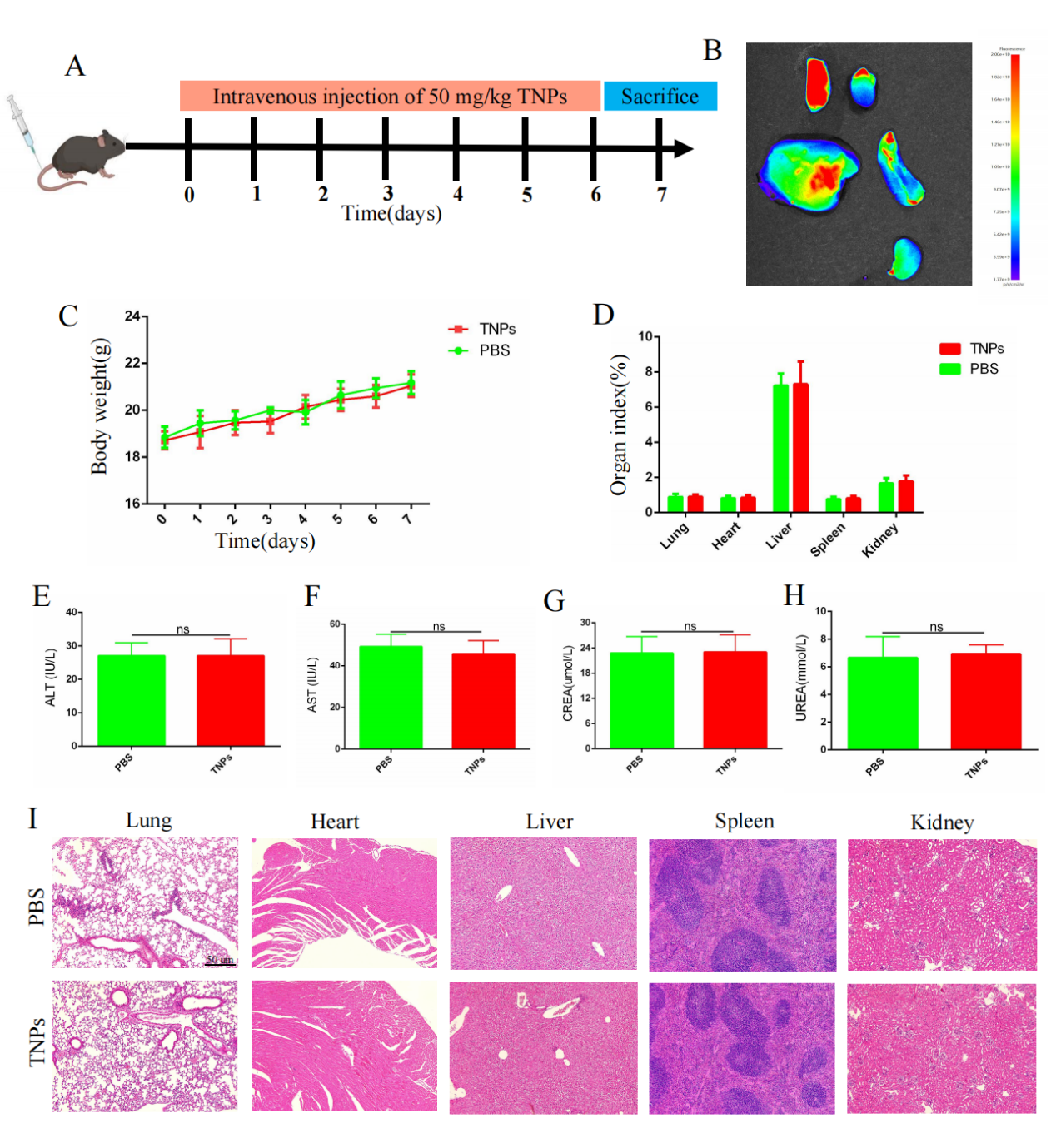
**

**Figure S4.** Toxicity evaluation of CoQ10@TNPs in mice. (A) Schematic diagram of experimental schemes. (B) CoQ10@TNPs distribution in the organs of the body. (C) Fluctuations in the body weight after i.v. injection of CoQ10@TNPs. (D) The major organs index after i.v. injection of CoQ10@TNPs. (E-H) Biochemical indicators associated with liver and kidney functions. (I) HE staining of typical major organs after i.v. injection of CoQ10@TNPs. Data are represented as mean ± SD (n = 4). ns: no significance.

**
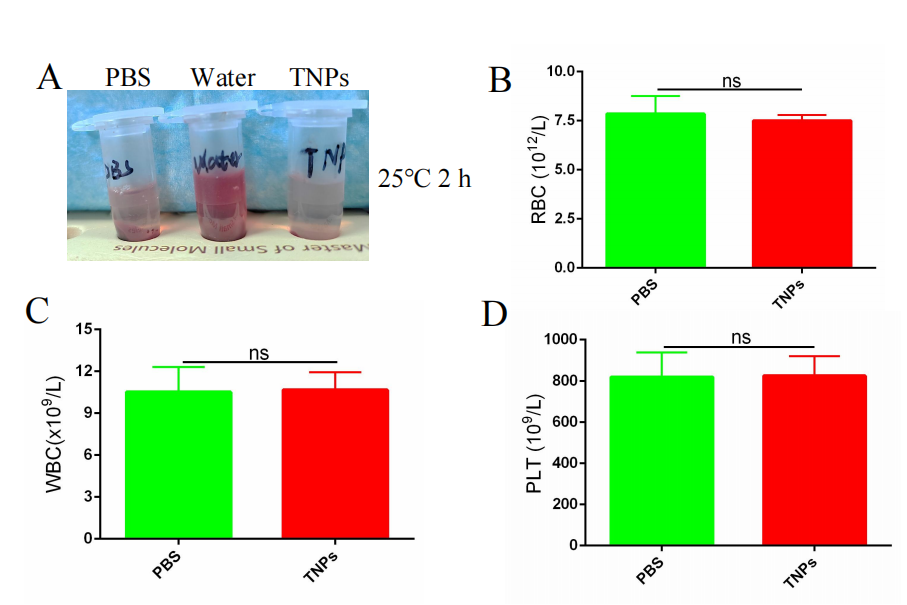
**

**Figure S5.** Blood compatibility of CoQ10@TNPs. (A) Hemolysis evaluation of CoQ10@TNPs in fresh erythrocytes. (B-D) The blood levels of WBC, RBC, and PLT after i.v. injection of CoQ10@TNPs. Data are represented as mean ± SD (n = 4). ns: no significance.


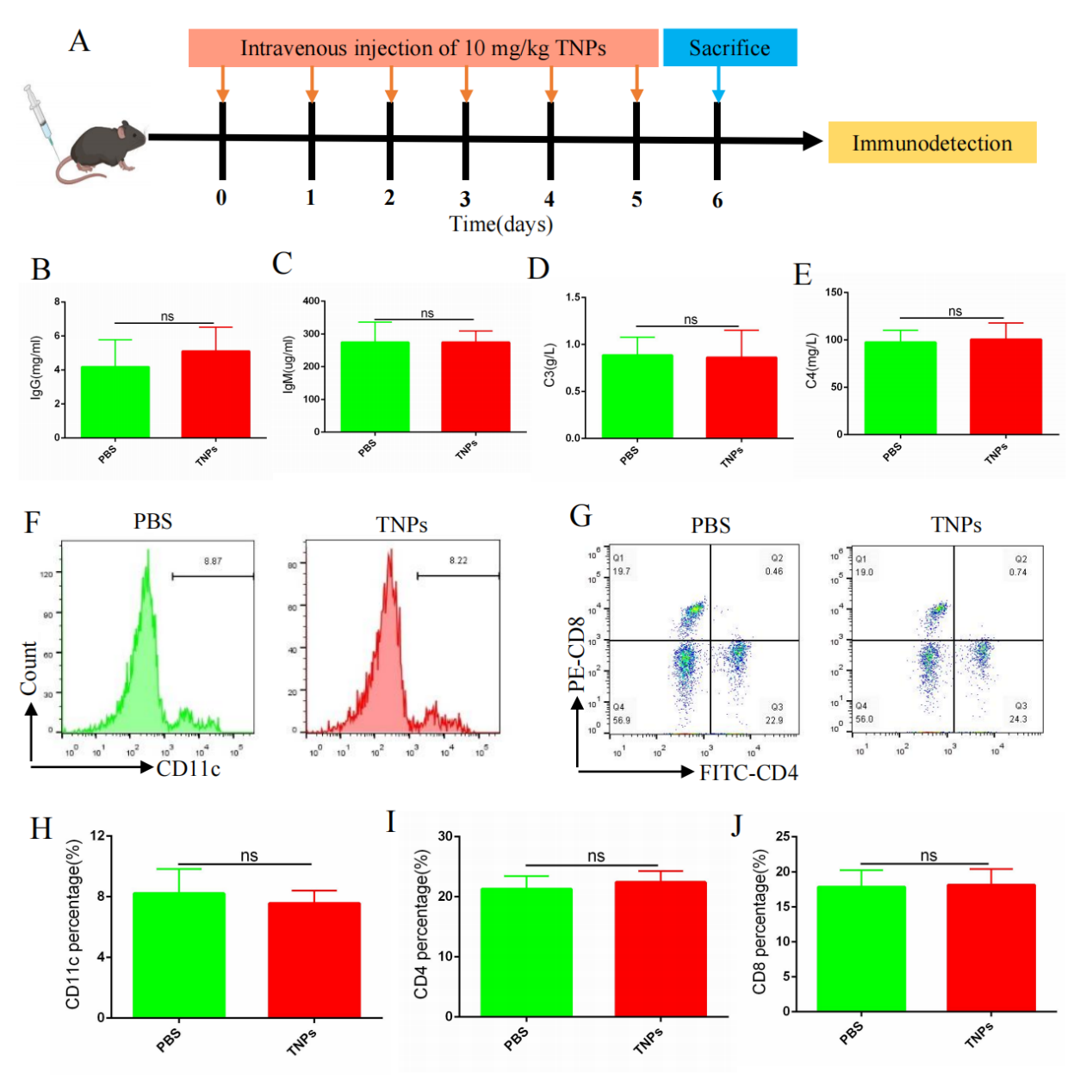


**Figure S6.** The immunogenicity examination of CoQ10@TNPs. (A) Schematic diagram of experimental schemes. (B, C) The expression levels of IgM and IgG in blood samples after multiple administrations. (D, E) The expression levels of C3 and C4 in blood samples after multiple administrations. (F, H) The proportion of CD11c+ immune cells after multiple administrations. (G, I, J) The proportion of CD4+ and CD8+ T cells after multiple administrations. Data are represented as mean ± SD (n = 4). ns: no significance.

**Figure S7.** CoQ10@TNPs reduce the activation of MAPK pathway. (A) Western blotting was employed to evaluate the protein levels of P-ERK, ERK, P-JNK, JNK, P-p38, p38.
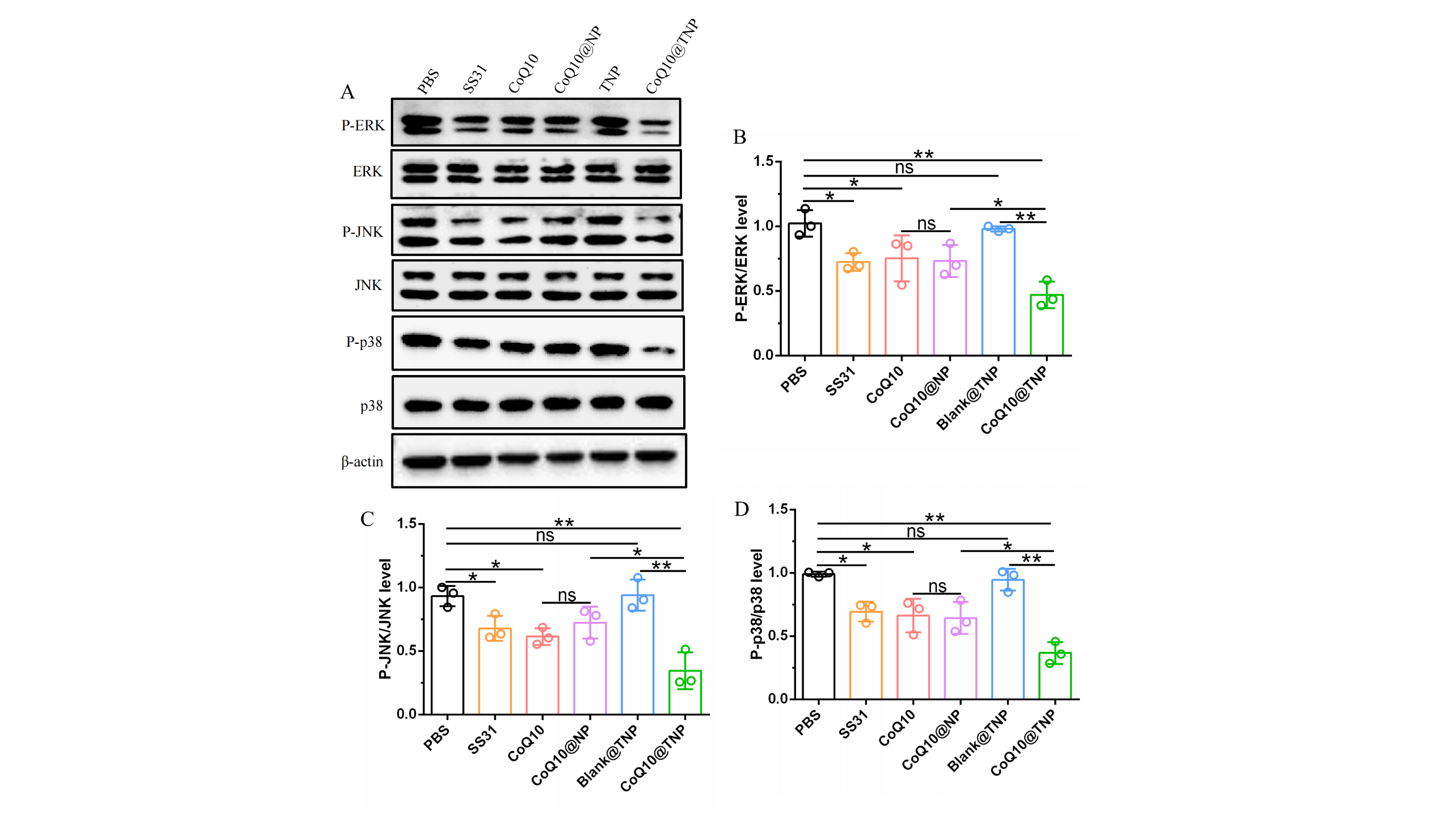
(B-D) Statistical analysis of P-ERK, P-JNK, P-p38. Data are represented as mean ± SD (n = 3). ns: no significance; *p < 0.05; **p < 0.01.
